# Determining Clinical Patient Selection Guidelines for Head and Neck Adaptive Radiation Therapy Using Random Forest Modelling and a Novel Simplification Heuristic

**DOI:** 10.3389/fonc.2021.650335

**Published:** 2021-06-07

**Authors:** Sarah Weppler, Harvey Quon, Colleen Schinkel, James Ddamba, Nabhya Harjai, Clarisse Vigal, Craig A. Beers, Lukas Van Dyke, Wendy Smith

**Affiliations:** ^1^ Department of Physics and Astronomy, University of Calgary, Calgary, AB, Canada; ^2^ Department of Medical Physics, Tom Baker Cancer Centre, Calgary, AB, Canada; ^3^ Department of Radiation Oncology, Tom Baker Cancer Centre, Calgary, AB, Canada; ^4^ Department of Oncology, University of Calgary, Calgary, AB, Canada; ^5^ Cumming School of Medicine, University of Calgary, Calgary, AB, Canada

**Keywords:** adaptive radiation therapy, head and neck cancer, patient selection guidelines, random forests, heuristics

## Abstract

**Purpose:**

To determine which head and neck adaptive radiotherapy (ART) correction objectives are feasible and to derive efficient ART patient selection guidelines.

**Methods:**

We considered various head and neck ART objectives including independent consideration of dose-sparing of the brainstem/spinal cord, parotid glands, and pharyngeal constrictor, as well as prediction of patient weight loss. Two-hundred head and neck cancer patients were used for model development and an additional 50 for model validation. Patient chart data, pre-treatment images, treatment plans, on-unit patient measurements, and combinations thereof were assessed as potential predictors of each objective. A stepwise approach identified combinations of predictors maximizing the Youden index of random forest (RF) models. A heuristic translated RF results into simple patient selection guidelines which were further refined to balance predictive capability and practical resource costs. Generalizability of the RF models and simplified guidelines to new data was tested using the validation set.

**Results:**

Top performing RF models used various categories of predictors, however, final simplified patient selection guidelines only required pre-treatment information for ART predictions, indicating the potential for significant ART process streamlining. The simplified guidelines for each objective predicted which patients would experience increases in dose to: brainstem/spinal cord with sensitivity = 1.0, specificity = 0.66; parotid glands with sensitivity = 0.82, specificity = 0.70; and pharyngeal constrictor with sensitivity = 0.84, specificity = 0.68. Weight loss could be predicted with sensitivity = 0.60 and specificity = 0.55. Furthermore, depending on the ART objective, 28%-58% of patients required replan assessment, less than for previous studies, indicating a step towards more effective patient selection.

**Conclusions:**

The above ART objectives appear to be practically achievable, with patients selected for ART according to simple clinical patient selection guidelines. Explicit ART guidelines are rare in the literature, and our guidelines may aid in balancing the potential clinical gains of ART with high associated resource costs, formalizing ART trials, and ensuring the reproducibility of clinical successes.

## Introduction

The spatial accuracy of IMRT and VMAT for head and neck radiotherapy can degrade over the course of treatment as tumor volumes and patient anatomy change. Previous studies in the literature indicate median decreases in gross tumor volume of 70% (1), and average weight loss of 8% (2) over the course of radical (chemo)radiotherapy. These anatomical changes may cause doses to organs-at-risk (OAR), such as the parotid glands, to increase in by >10 Gy (3), and target coverage to degrade by >5% (4) in select patients. Adaptive radiation therapy (ART) replans patient treatments in response to anatomical changes, with single-institution clinical trials showing that ART may improve 2-year local regional control by 9% (5), reduce xerostomia and dysphagia by an estimated 11% (6) and significantly improve post-treatment quality of life (7).

Treatment replanning is simple in concept, yet routine ART is hampered by practical constraints. Replanning all head and neck cancer patients can place a significant burden on dosimetry, medical physics, and other departments (8). In addition, only about 20% of patients are expected to benefit from replanning (3), however, criteria to effectively identify these patients have not yet been established in the literature. Current patient selection for treatment replanning is often subjective, according to clinician discretion, making it challenging to reproduce the above ART trial results and successes. Simple ART patient selection approaches, such as monitoring changes in a patient’s external contour, may be no better than randomly selecting patients for replanning (9). Existing ART models for patient selection show promise but still suffer from limited performance (10, 11).

In this study, we develop simple guidelines to select patients for ART (including physician/physicist review of delivered doses, re-CT, refitting of immobilization, and/or treatment replanning), with the objective of decreasing the likelihood of toxicity, poor post-treatment quality of life, and/or tumor recurrence. We use random forest (RF) models to examine which ART objectives are practically achievable (i.e., predictable with reasonable resource use, according to RF capabilities), and further simplify model results using a novel heuristic to develop clinical patient selection guidelines. While full RF models capture the complexity of predictor-response associations, heuristic-based guidelines are more transparent and of a format that is familiar and intuitive for clinical staff. Our hope is that this step towards explicit ART patient selection guidelines will fill an important gap in the ART literature, allow for the formalization of ART trials and improve the reproducibility of clinical ART studies. Furthermore, such a modelling-simplification paradigm as presented in this study is generalizable to a variety of clinical settings that strive to balance the insight gained from complex analyses with the clarity required for clinical implementation.

## Materials and Methods

### Patient Inclusion Criteria

The study cohort consisted of 250 head and neck cancer patients treated at a single center with radical VMAT (chemo)radiotherapy (70 Gy/33 fractions) between November 2015 and September 2018. The VMAT technique used 2 arcs of 6 MV photons. Radiotherapy treatment planning objectives for planning target volumes (PTVs) and OAR are provided in **Table 1**. Patient radiotherapy treatments were planned using the Eclipse Treatment Planning System, Versions 11 and 13 (Varian Medical Systems, Palo Alta, CA). Institutional image-guided radiation therapy protocols used daily kV-orthogonal imaging and weekly kV-cone beam CT (CBCT) imaging. This study was approved by our institutional research ethics board (HREBA.CC-18-0093).

**Table 1:** Radiotherapy treatment planning objectives.

Structure Type	Planning Objective
Target	High-dose PTV D95% ≥ 70 Gy
	High-dose PTV D2% ≤ 77 Gy
	Low-dose PTV D95% ≥ 59.4 Gy
	Low-dose PTV D20% ≤ 65.3 Gy
Organs-at-Risk	Brainstem D0.03cc ≤ 54 Gy
	Spinal cord D0.03cc ≤ 48 Gy
	Pharyngeal constrictor Dmean ≤ 50 Gy
	Ipsilateral and contralateral parotid gland Dmean ≤ 26 Gy
	Ipsilateral and contralateral submandibular gland Dmean ≤ 39 Gy

### Potential Predictors

**Table 2** lists potential predictors identified based on clinical experience and according to measures broadly suggested in the literature. These have been collected from the patients’ electronic medical record (EMR), contoured planning CT (pCT), treatment plan (RTx), and rigid alignments of planning CT and last-acquired on-unit CBCT images (Obs). Some measurements, such as changes in brainstem and spinal cord volume, were included to identify errors in deformable image registration (DIR) image processing, as volumetric changes in these structures with progression through treatment is not expected. **Supplementary Material – Part 1** provides further details of CT-CBCT measurements.

**Table 2:** Input data and categories used for RF model development.

Patient and Tumor Data from Electronic Medical Record (EMR)	Planning CT Data (pCT)	Treatment Plan Data (RTx)	Patient Monitoring and CBCT-Based Measurements (Obs)*
Age	Structure volumes at planning:	Planned dose parameter values:	ΔFace diameter
Gender	* High-dose CTV	* High-dose CTV D95%, D2%	ΔNeck diameter
Cancer Site	* Low-dose CTV	* Low-dose CTV D95%, D20%	ΔNeck/shoulder contour
TNM Stage	* Brainstem	* Brainstem D0.03cc	Head rotation
Chemotherapy agent	* Spinal cord	* Spinal cord D0.03cc	Chin tilt
ECOG performance status	* Pharyngeal constrictor	* Pharyngeal constrictor Dmean	ΔShoulder position
Charlson comorbidity index	* Ips./cont. parotid gland	* Ips./cont. parotid gland Dmean	ΔBMI
HPV status	* Ips./cont. submandibular gland	* Ips./cont. submandibular gland Dmean	Percutaneous endoscopic gastrostomy or nasogastric tube placement
Smoking history			
Drinking history			
Initial BMI			
Disease laterality			
Bolus			

Ips., ipsilateral; Cont., contralateral; Δ, change relative to value at planning.

*See **Supplementary Material – Part 1** for measurement details.

### Adaptive Radiation Therapy Objectives

We independently considered nine ART objectives of interest, where initial RF models were developed to predict which patients would experience:

1. Increases in brainstem/spinal cord Dmax (whichever structure was planned closer to or farther exceeded the planning objective) - potentially increasing the risk of brainstem necrosis or myelopathy;
2. Increases in parotid gland Dmean for the gland planned with the lowest mean dose - potentially increasing the risk of xerostomia;
3. Increases in pharyngeal constrictor Dmean – potentially increasing the risk of dysphagia;
4. Increases in submandibular gland Dmean for the gland planned with the lowest mean dose – potentially increasing the risk of xerostomia;
5. Decreases in high-dose CTV D95% target coverage – potentially increasing the risk of tumor recurrence;
6. Increases in high-dose CTV D2% target hotspot – potentially increasing the risk of tissue necrosis;
7. Increases in volume of high-dose CTV - potentially indicating poor treatment response;
8. Decreases in body mass index (BMI) – potentially prognosticating poorer overall survival and disease-specific survival;
9. Increases in on-unit patient setup time from the first kV-orthogonal image to beam-on, including CBCT-based adjustments – indicating greater staffing and resource costs.

Although objectives are expected to be correlated, each RF model was developed to predict a specific objective in an attempt to clarify predictor-objective associations. Further detail on the clinical implications of select objectives is provided in **Table 3**.

**Table 3:** Objectives, normal/violation deviation tolerances, and potential clinical implications of violations.

ART Objective	Definition	Tolerance on planning criteria violation or ALARA deviation from planned value* (% patients with violation)		Implications of Objective Violations on Toxicity and Clinical Outcomes
		Trend Analysis	Quartile	
Brainstem/spinal cord	Brainstem D0.03cc ≥ 54 Gy OR spinal cord D0.03cc ≥ 45 Gy)	1.1 Gy (20%)	0.8 Gy	Increased risk of severe or permanent neurological effects (12); >0.03% risk of myelopathy increasing to 0.2% at 50 Gy (13); V45 Gy > 14.15 cc for Lhermitte sign (14); increase in total MFI-20 acute patient fatigue scores of 0.3 over baseline per 1 Gy (15);
Parotid glands	Ips. AND cont. parotid gland Dmean ≥ 26 Gy	2.2 Gy (27%)	0.9 Gy	Little or no recovery of stimulated salivary flow (16); increase in the risk of grade 2 or worse xerostomia by 20% for each 1 Gy over 26 Gy (17); decrease in long-term salivary function to <25% for doses >25 Gy (18);
Pharyngeal constrictor	Pharyngeal constrictor Dmean ≥ 50 Gy	0.8 Gy (47%)	1.5 Gy	>20% risk of dysphagia (19, 20); increase in the risk of dysphagia by 19% per 10 Gy after 55 Gy (19); decreased QoL scores in speech and social function (21)
Weight loss	During-treatment decrease in BMI (quartile of patients with greatest weight loss)	1.83 kg/m^2^ (68%) (or average weight loss ≥ 6.8%)	3.4 kg/m^2^ (or average weight loss ≥12.8%)	Decreases in five-year overall survival of 8% and decreases in disease-specific survival of 7% for >10% weight loss (22); >10% weight loss had a significant impact on quality of life (23)

MFI-20, Multidimensional Fatigue Inventory; *Based on equations (1) and (2) for dosimetric objectives.

An inter-fractional anatomic or dosimetric change potentially increasing the risk of an adverse effects is defined as a “violation” warranting an ART replan assessment. All other changes were considered “normal” (e.g., resulting from minor anatomical changes or variations in patient setup).

#### Dosimetric ART Objectives

##### Deformable Image Registration Workflow Quality Assurance

Delivered dose was estimated by deformably registering the planning CT and last-acquired CBCT images (Velocity™ Version 3.2.0, Varian Medical Systems) (24, 25), copying the original treatment plan to the resulting contoured “synthetic CT”, and recalculating dose (26). Therefore, synthetic CTs combined the clinician contours, field of view, and HU calibration curve of the planning CT with changes in anatomy captured by the last-acquired on-unit CBCT. Quality assurance of the workflow compared DIR output with the consensus contours of two radiation oncologists specializing in head and neck cancer, on a subset of representative images (27–29). Full details on the quality assurance analysis approach is provided in **Supplementary Material – Part 2**.

##### Patient Data Labels: Normal vs. Violation

To formalize normal *vs.* violation labels for each patient, according to each objective, we established tolerances to distinguish random variations (i.e., resulting from daily setup changes or workflow error) from systematic dose degradations. For this, we additionally analyzed the weekly CBCTs for 10 patients randomly selected from the cohort (65 synthetic CTs), performed a linear fit to each patient’s weekly trend data (given the noise in trend data), and calculated the difference between the linear trend and actual objective estimate based on the last-acquired CBCT. Twice the standard deviation of these differences across all patients provided a random error deviation tolerance; violations in objective values exceeding the deviation tolerance were more likely to result from systematic effects.

Given the deviation tolerance, we first determined normal *vs.* violation labels according to “planning criteria violations”. For patients with planned doses *meeting* planning criteria, violations were present if:

(1) delivered dose≥planning criteria+deviation tolerance

For patients with planned doses *exceeding* planning criteria, violations were present if:

(2) delivered dose≥planned dose+deviation tolerance

Secondly, we considered an “as low as reasonably achievable” (ALARA) screening paradigm that applied equation (2) to all patients, correcting, for example, any dose increases above planned values, including consideration of the deviation tolerance.

For comparison, for each of the planning criteria violations and ALARA approaches, we identified the quartile of patients with the worst planning criteria and ALARA violations without consideration of these random/systematic tolerances. Therefore, for each endpoint, we considered four normal/violation formats (planning criteria violations + deviation tolerance; ALARA + deviation tolerance; planning criteria violations + poorest quartile; ALARA + poorest quartile). Additional details and examples of the planning criteria and ALARA violation definitions may be found in **Supplementary Material – Part 3**.

#### Clinical and Volumetric ART Objectives

Changes in the volume of the high-dose CTV were calculated from planning and synthetic CTs. Clinical and volumetric objectives had no planning objectives or pre-defined tolerances. Instead, we calculated the deviation tolerance of linearly projected trend values *vs.* calculated values to give a sense of the relative contribution of random noise in the data. For RF model development, we identified the quartile of patients with the most unfavorable relative changes in objective values (ALARA + poorest quartile formatting).

### Analysis

#### Training and Validation Datasets

We developed RF models using the first 200 chronological patients (treated November 2015 – January 2018). The subsequent 50 patients (treated January 2018 – September 2018) were reserved for model validation. Cohort characteristics are summarized in **Table 4**.

**Table 4:** Cohort demographic and clinical characteristics.

Parameter	Full Cohort (n = 250)	Cohort for Model Development (n = 200)	Cohort for Validation (n = 50)
Age in years, mean (±SD)	58.7 (10.1)	58.6 (10.3)	58.9 (9.4)
Gender, number (%)			
Male	221 (88.4%)	174 (87.0%)	47 (94.0%)
Female	29 (11.6%)	26 (13.0%)	3 (6.0%)
Initial BMI, mean (±SD)	27.6 (5.8)	27.6 (5.8)	27.7 (5.6)
ECOG, median (range)	1 (0-3)	0 (0-3)	1 (0-3)
Charlson Comorbidity Index, median (range)	4 (1-9)	4 (1-7)	4 (2-9)
Alcohol use, number (%)			
Never	55 (22.0%)	45 (22.5%)	10 (20.0%)
Former	18 (7.2%)	14 (7.0%)	4 (8.0%)
Current – Light (males 0-15 drinks/week, females 0-10 drinks/week)	127 (50.8%)	103 (51.5%)	24 (48.0%)
Current – Heavy (males >15 drinks/week, females >10 drinks/week)	50 (20.0%)	38 (19.0%)	12 (24.0%)
Tobacco use, number (%)			
Never	93 (37.2%)	73 (36.5%)	20 (40.0%)
Cumulative – Light (0-20 pack-years)	71 (28.4%)	60 (30.0%)	11 (22.0%)
Cumulative – Heavy (>20 pack-years)	86 (34.4%)	67 (33.5%)	19 (38.0%)
Primary tumor location, number (%)			
Larynx	22 (8.8%)	14 (7.0%)	8 (16.0%)
Hypopharynx	9 (3.6%)	7 (3.5%)	2 (4.0%)
Oral Cavity	20 (8.0%)	17 (8.5%)	3 (6.0%)
Oropharynx	145 (58.0%)	117 (58.5%)	28 (56.0%)
Nasal Cavity	7 (2.8%)	7 (3.5%)	0 (0.0%)
Nasopharynx	36 (14.4%)	28 (14.0%)	8 (16.0%)
Unknown	11 (4.4%)	10 (5.0%)	1 (2.0%)
T stage, number (%)			
T0 – T2	119 (47.6%)	96 (48.0%)	23 (46.0%)
T3 – T4	110 (44.0%)	85 (42.5%)	25 (50.0%)
Tis	1 (0.4%)	1 (0.5%)	0 (0.0%)
Tx	20 (8.0%)	18 (9.0%)	2 (4.0%)
N stage, number (%)			
N0	34 (13.6%)	27 (13.5%)	7 (14.0%)
N1	30 (12.0%)	14 (7.0%)	16 (32.0%)
N2	164 (65.6%)	146 (73.0%)	18 (36.0%)
N3	19 (7.6%)	10 (5.0%)	9 (18.0%)
NX	3 (1.2%)	3 (1.5%)	0 (0.0%)
p16 status, number (%)			
Negative	49 (19.6%)	31 (15.5%)	18 (36.0%)
Positive	153 (61.2%)	126 (63.0%)	27 (54.0%)
Unknown	48 (19.2%)	43 (21.5%)	5 (10.0%)
Radiotherapy treatment, number (%)			
Unilateral	20 (8.0%)	16 (8.0%)	4 (8.0%)
Bilateral	230 (92.0%)	184 (92.0%)	46 (92.0%)
Chemotherapy agent, number (%)			
Capecitabine (Xeloda)	5 (2.0%)	5 (2.5%)	0 (0.0%)
Carboplatin	20 (8.0%)	18 (9.0%)	2 (4.0%)
Cetuximab	38 (15.2%)	36 (18.0%)	2 (4.0%)
Cisplatin (Cisplatinum)	176 (70.4%)	135 (67.5%)	41 (82.0%)
None	11 (4.4%)	6 (3.0%)	5 (10.0%)

#### Random Forest Modelling

Random forest models were selected for their predictive capability and versatility (30), as well as analogy to clinical decision-making paradigms. Conceptually, these algorithms look at the majority vote of a set of decision trees, similar to an assessment by multiple clinicians.

**Figure 1:**
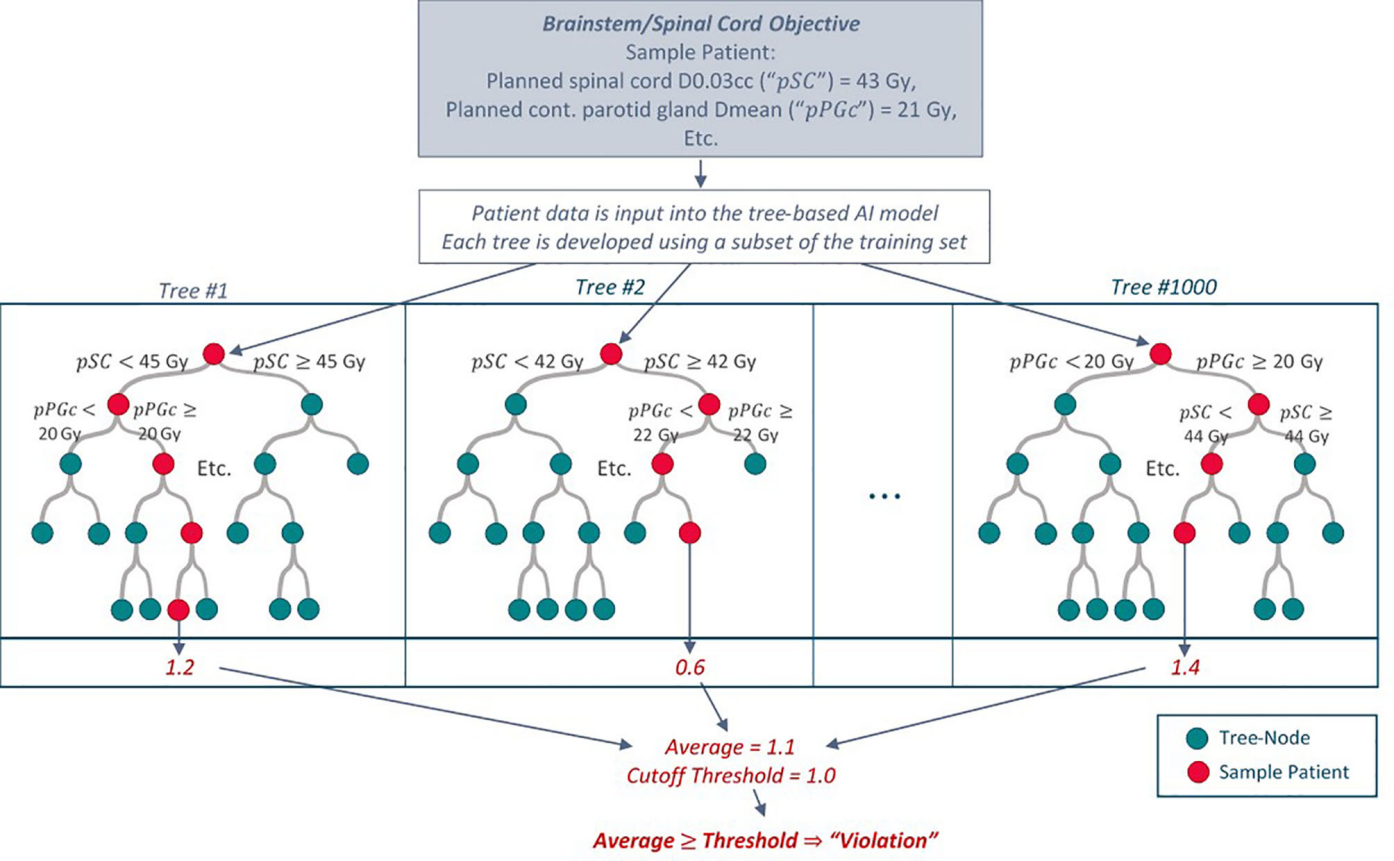
Schematic of how the tree-based RF models predict an ART objective violation for a given patient with “toy” values for illustration purposes. Each tree within the model is developed using a random subset of patients in the training dataset. Additional specifications are placed on how each tree is grown (only a random subset of predictors is available to split upon at each tree node). To predict an objective violation for a new patient, patient data is input into the model. An average violation estimate from all trees indicates whether the patient may require a replan assessment.

The RF models used all predictor categories (EMR, pCT, RTx and Obs in **Table 2**) and combinations of categories to predict the magnitude of a violation for each objective except for #8: decreases in BMI. RF models for the latter excluded the Obs predictor category (already containing ΔBMI) and used only pre-treatment data (EMR, pCT, RTx). As RF model initialization is stochastic in nature, we used five different random initializations for each model. Receiver operating characteristic (ROC) curves were produced for each model and initialization by incrementally varying the value (“threshold”) required to convert a five-fold cross validated numerical violation estimate (regression) to categorial normal/violation output. A schematic for the prediction of violations using a trained RF model and a sample “toy” input is shown in **Figure 1**. The point on the ROC curve maximizing the sum of sensitivity and specificity (i.e., maximum Youden index) served as the primary metric for assessing model performance for a given ART objective. Area under the curve (AUC) provided additional information on model performance.

To identify which objectives were most predictable given all combinations of potential predictor sets (EMR, pCT, RTx, Obs) and reference normal/violation paradigms (planning criteria and ALARA violations, with deviation tolerances or poorest quartile), we used a greedy stepwise approach (31) and Kruskal-Wallis rank-sum tests. Such an approach identified top performing RF models to be heuristically refined to produce simple patient selection guidelines. For each objective, parameters that most clearly differentiated models with strong *vs.* poor predictive capability according to Youden index were selected first; this parameter was then fixed and the process repeated. When multiple combinations of predictors produced ROC curves with a similar Youden index, we identified the model with the largest set of input parameters (most complete) and the model with the smallest set of input parameters (most parsimonious) for further testing. Of these two, the model obtaining a higher specificity for sensitivity values ranging from 0.60-0.80 was selected. Further details of RF model development and selection is included in **Supplementary Material – Part 4**.

While our sample size is relatively large for ART predictive model development, it is fairly small in the field of machine learning. To consider how sample size may have affected model performance, we further developed models using the first 100, 125, 150 and 175 consecutive patients from the training cohort and assessed five-fold cross validated estimates of sensitivity and specificity.

#### Heuristic to Derive Simplified Patient Selection Guidelines for ART

**Figure 2:**
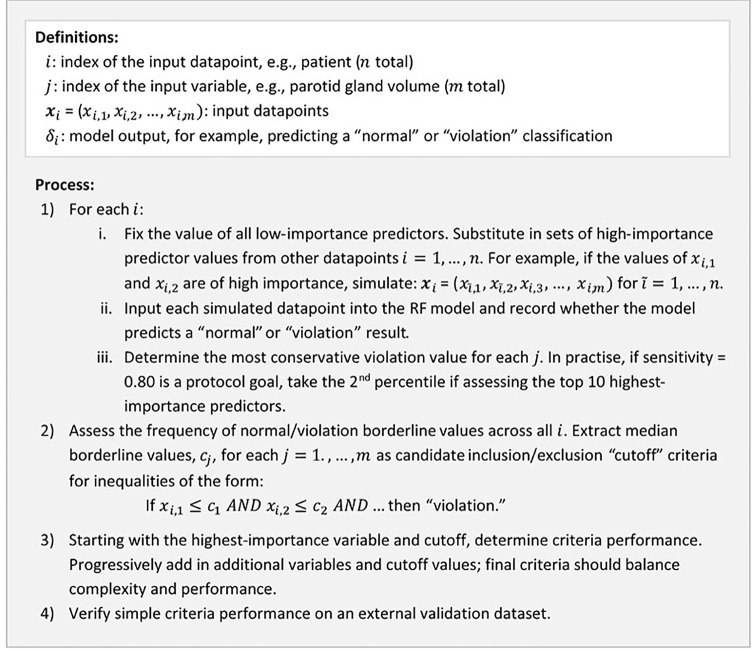
Summary of the heuristic process used to convert RF model results into simple ART patient selection guidelines.

To derive simple patient selection guidelines from the RF models, we modified an existing heuristic approach (26). Details of the present heuristic process are provided in **Figure 2**. Conceptually, RF models are simplified by determining the values of high-importance predictors (according to mean squared error on out-of-bag samples) at the boundary of normal *vs.* violation predictions. Combinations of predictor values producing boundary results provided “cutoff” guidelines for patient selection. An explicit example of this heuristic process for the ART parotid gland sparing objective is presented in **Supplementary Material – Part 5**.

**Figure 3:**
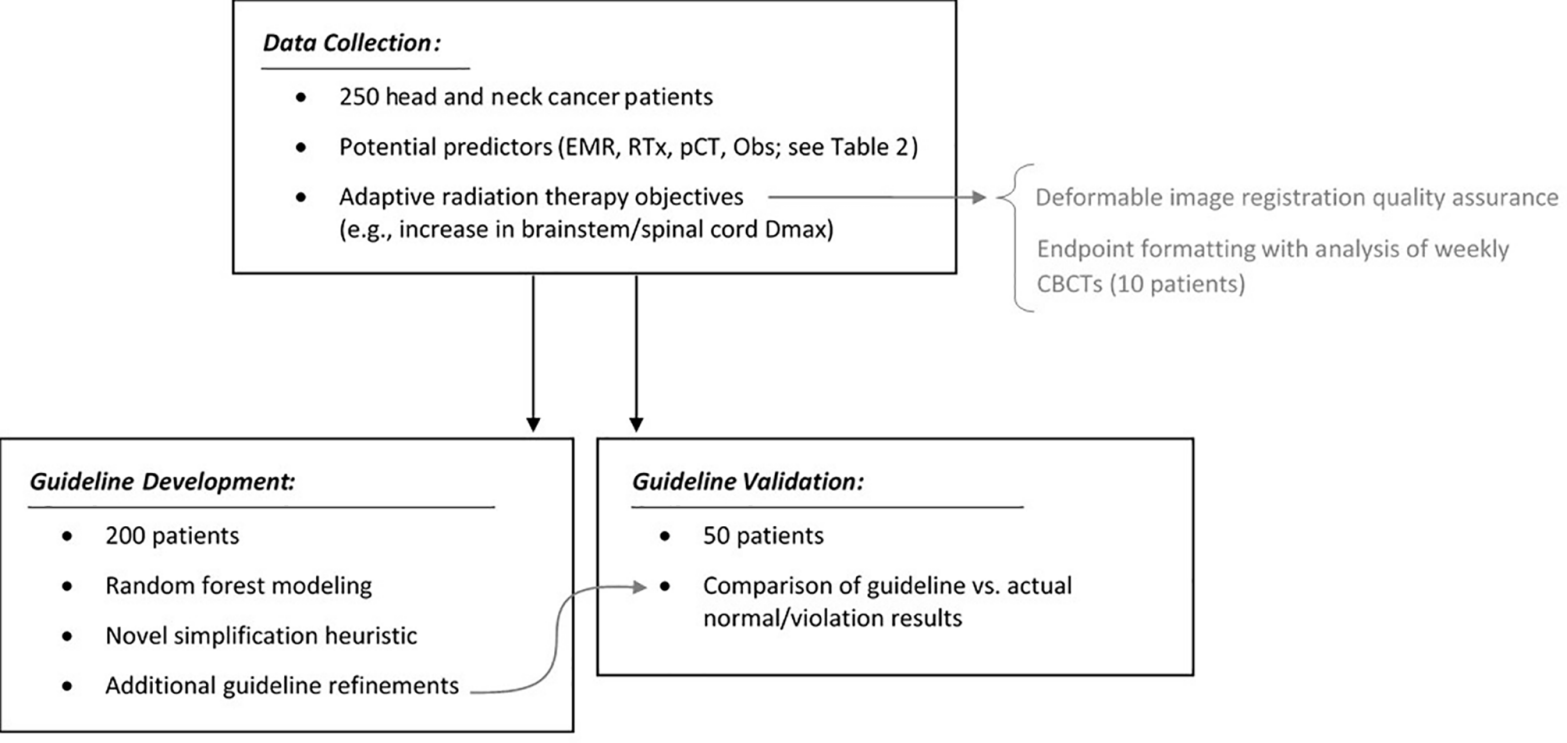
Summary of the study design: data collection, auxiliary analyses, guideline development, and guideline validation.

**Figure 3** summarizes the study design with respect to data collection, guideline development, and guideline validation. All analyses were performed in R (R Version 3.5.1, The R Foundation for Statistical Computing, Vienna, Austria) using the base and randomForest libraries.

## Results

**Figure 4:**
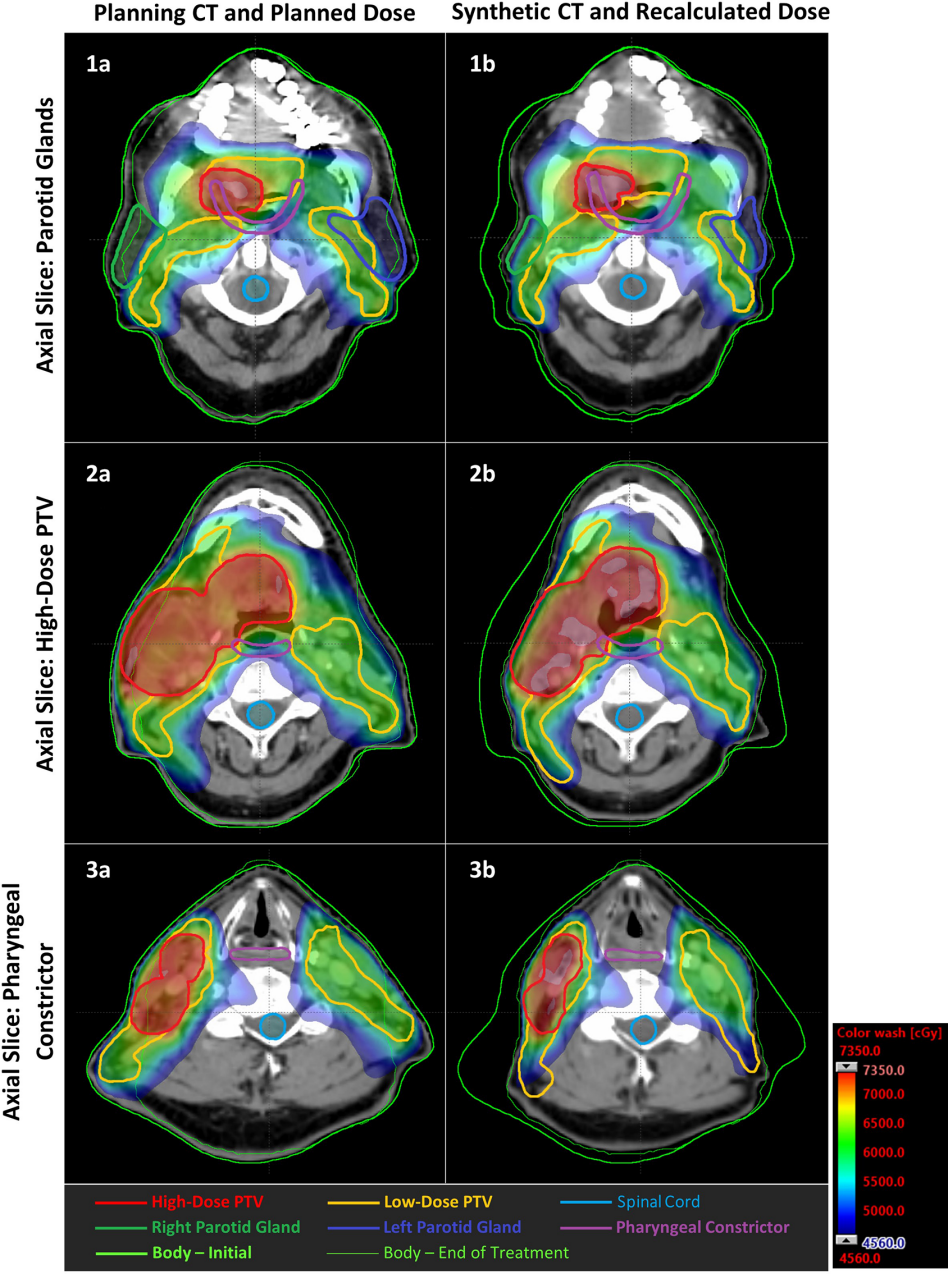
Example of the changes in patient geometry and dosimetry between the planning CT (**A**: left column) and synthetic CT (**B**: right column), here assessed at fraction 31 of 33. The patient shown was identified as having changes representative of approximately 12% of the training cohort, according to data clustering performed for deformable image registration quality assurance. Axial slices correspond to: 1) the centers of mass of the parotid glands; 2) centre of mass of the high-dose PTV; 3) centre of mass of the pharyngeal constrictor, assessed for the planning CT and rigid alignment of the synthetic CT. A dose color wash indicates doses ranging from 95% of the maximum allowable spinal cord dose, to 105% of the high-dose prescription. Anatomical structure contours are overlaid. Notably, the patient experienced weight loss, loss of parotid gland volume, and a general increase in doses to healthy tissues.

**Figure 4** provides a representative example of the geometric and dosimetric changes in patient anatomy occurring between the planning CT and synthetic CT.

### Dosimetric ART Objectives

#### Deformable Image Registration Quality Assurance

DIR and physician contours were geometrically (27) and dosimetrically (28) consistent for all except two anatomical structure types (**Supplementary Material – Part 2**), validating the DIR workflow used. Exceptions were submandibular glands and high-dose CTV target coverage; as a result, RF models were not developed for the corresponding ART objectives.

#### Patient Data Labels: Normal *vs.* Violation

Deviation and quartile tolerances from the trend analysis are included in **Table 3** for select ART objectives. Omitted from **Table 3** is patient setup time, which did not show systematic trends with progression through treatment. In addition, only 6 of 250 patients had increases in high-dose CTV D2% exceeding planning criteria, creating a dataset with low prevalence. Both setup time, and CTV D2% objectives were omitted from RF model development. Further details on deviation and quartile tolerances are provided in **Supplementary Material – Part 3**.

### Random Forest Modelling

**Figure 5:**
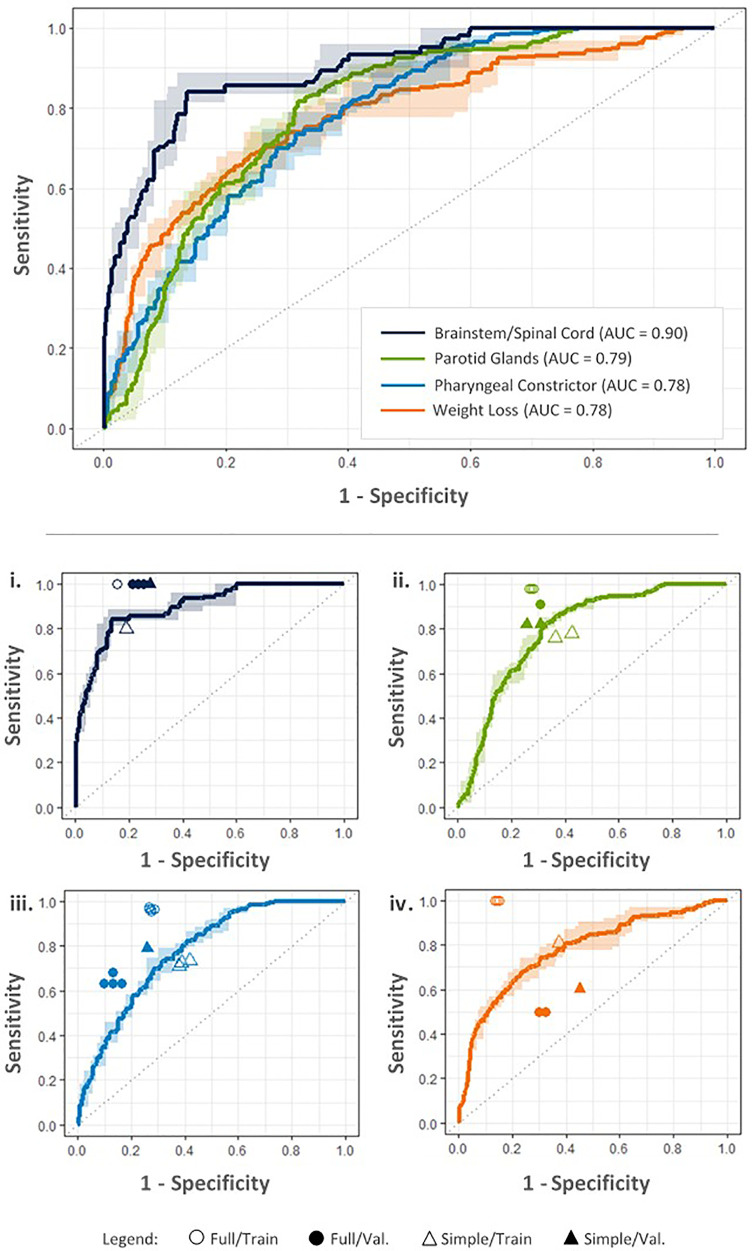
ROC curves for each objective based on the best performing RF models according to maximum Youden index, produced using input parameters indicated in the **Table 5**. Upper: ROC curves estimate tradeoffs in model sensitivity and specificity using five-fold cross validation on the training dataset. Dark lines denote average model performance across five random model initializations; average AUC is included in the legend. Corresponding ranges in model sensitivity and specificity are indicated by light colored bands. Lower: Performance of final full RF models on the training (Full/Train) and external validation datasets (Full/Val.) is compared with simplified criteria performance (Simple/Train, Simple/Val.) for i. brainstem/spinal cord Dmax, ii. parotid gland Dmean, iii. pharyngeal constrictor Dmean, and iv. decrease in BMI (weight loss) objectives.

**Table 5** summarizes the achievability and predictor sets required for each of the ART objectives. Of these, for RF models achieving AUC≥0.75, **Figure 5** shows ROC curves averaged over the five random initializations.

**Table 5:** Simplified patient selection guidelines for ART based on the most predictive RF models.

Objective	(1) Can the objective be predicted?*	(2) Which data are required for model predictions?	(3) Can RF models be simplified and patient selection streamlined?	Simple Patient Selection Criteria†
1) Increase in brainstem/spinal cord Dmax	Yes. AUC = 0.90	RTx, Obs	Yes	If Planned brainstem D0.03cc ≥ 16 Gy
	(Sensitivity = 1.0, Specificity = 0.77)			AND Planned cont. parotid gland Dmean ≥ 20 Gy
				AND Planned cont. submand. gland Dmean ≥ 34 Gy
				AND Planned ips. parotid gland Dmean ≥ 25 Gy
				AND Planned pharyngeal constrictor Dmean ≥ 45 Gy
				AND Planned spinal cord D0.03cc ≥ 43 Gy
				then violation likely.
				(Sensitivity = 1.0, Specificity = 0.66)
2) Increase in parotid gland Dmean	Yes. AUC = 0.79	RTx	Yes	If Planned brainstem D0.03cc ≥ 16 Gy
	(Sensitivity = 0.91, Specificity = 0.69)			AND Planned cont. parotid gland Dmean ≥ 24 Gy
				AND Planned cont. submand. gland Dmean ≥ 33 Gy
				AND Planned ips. parotid gland Dmean ≥ 24 Gy
				AND Planned ips. submand. gland Dmean ≥ 61 Gy
				AND Planned low-dose CTV D20% ≥ 64 Gy
				AND Planned pharyngeal constrictor Dmean ≥ 45 Gy
				AND Planned spinal cord D0.03cc ≥ 41 Gy
				then violation likely.
				(Sensitivity = 0.82, Specificity = 0.70)
3) Increase in pharyngeal constrictor Dmean	Yes. AUC = 0.78	EMR, pCT, RTx, Obs	Yes	If Planned brainstem D0.03cc ≥ 16 Gy
	(Sensitivity = 0.64, Specificity = 0.87)			AND Planned cont. parotid gland Dmean ≥ 19 Gy
				AND Planned cont. submand. gland Dmean ≥ 34 Gy
				AND Planned ips. parotid gland Dmean ≥ 21 Gy
				AND Planned pharyngeal constrictor Dmean ≥ 49 Gy
				AND Planned spinal cord D0.03cc ≥ 40 Gy
				AND Initial low-dose CTV volume ≥ 197cc
				then violation likely.
				(Sensitivity = 0.84, Specificity = 0.68)
4) Increase in submandibular gland Dmean	No (excess geometric error arising from DIR workflow)			–
5) Decrease in high-dose CTV D95%	No (excess dosimetric error arising from DIR workflow)			–
6) Increase in high-dose CTV D2%	No (too few patients with violation to produce a predictive model)			–
7) Increase in volume of high-dose CTV	Weakly.‡	Obs	No (model performance not strong enough)	–
	AUC = 0.63			
	(Sensitivity = 0.75, Specificity = 0.47)			
8) Decrease in patient BMI (weight loss)	Yes. AUC = 0.78	EMR, pCT, RTx	Yes	If Initial BMI ≥ 27 kg/m^2^
	(Sensitivity = 0.50, Specificity = 0.70)			then violation likely.
				(Sensitivity = 0.60, Specificity = 0.55)
9) Increase in on-unit patient setup time	No (random interfractional changes dominate systematic effects)			–

Sensitivity and specificity correspond to values obtained on the validation dataset. *Model performance based on the training point maximizing Youden index, averaged over the five random model initializations. ^†^Predictive performance of simple guidelines on the validation dataset. ^‡^Attributed to borderline geometric acceptability of DIR output.

In general, factors most affecting model performance included: predictor set combinations (EMR, pCT, RTx, and/or Obs), followed by normal/violation formatting (planning criteria *vs.* ALARA violation; deviation tolerance *vs.* poorest quartile). Models based on planning criteria violations outperformed those based on the ALARA paradigm. Furthermore, for dosimetric objectives, models developed using deviation tolerances outperformed those identifying the quartile of patients with the largest violations.

Youden index decreased for the validation dataset, as expected, with an average decrease across all objectives of 0.12. This behavior generally occurs due to slight model overfitting on training data (10).

Constraints on training cohort size did not appear to limit RF model results. Average AUC only increased by 1% when doubling the size of the training dataset from 100 to 200 patients. However, the standard deviation of AUC for the five random initializations of each model decreased by an average of 44%.

### Heuristically Simplified Patient Selection Guidelines

**Table 5** gives the simple patient selection guidelines and performance on the validation dataset for the achievable ART objectives. The percentages of patients indicated for replan assessment were: 28% for brainstem/spinal cord; 33% for parotid glands; 58% for pharyngeal constrictor; and 49% for weight loss. For the simplified criteria, Youden index on the validation dataset increased by an average of 0.15 compared to the training dataset.

Although some of the top performing models included elements from the EMR and Obs input categories, these could be removed from the simplified criteria with only minor losses in sensitivity and specificity. For the brainstem/spinal cord Dmax objective, ΔNeck diameter ≥5mm was originally included in the patient selection criteria. For the pharyngeal constrictor Dmean objective, the heuristic retained ΔFace diameter ≥6mm and bilateral treatment. For the latter, all patients planned with a contralateral parotid gland Dmean exceeding 19 Gy received bilateral treatment, and the redundant EMR parameter was removed. Furthermore, removing the on-unit measurements (ΔNeck diameter, ΔFace diameter) reduced specificity by 0.06 for both brainstem/spinal cord and pharyngeal constrictor objectives. The moderate reduction in performance may have significant gains in overall ART workflow streamlining as further examined below.

## Discussion

This study shows that RF modelling may be used to examine complex data associations, where results may be heuristically simplified to produce clinical guidelines for clinicians that are familiar and intuitive. Previous studies have aimed to predict various ART objectives (10, 11, 32, 33). While a comparable model in the literature predicting parotid gland dose increases achieved specificity of 0.25 for sensitivity of 0.80 on a validation dataset (10), our models and simplified guidelines have achieved promising specificity of approximately 0.70 (sensitivity ≥0.80). In addition, our ART patient selection targets a smaller number of patients for replan assessment (28-58%) compared to 58% to 77% for parotid gland objectives previously published (10, 32). Combining patient selection criteria from our study for brainstem/spinal cord, parotid gland, and pharyngeal constrictor objectives corresponds to ART referral for 65% of patients. While replanning 65% of patients may currently be too resource costly for rollout in busy clinics, the cost-benefit tradeoff for brainstem/spinal cord or parotid gland sparing may be more feasible. It may be possible to further refine pharyngeal constrictor and weight loss models by evaluating modified objective criteria (e.g., besides Dmean ≥50 Gy), although this falls outside of the scope of the present work and QUANTEC-motivated constraints.

By removing on-unit measurements in the simplified patient selection criteria, ART workflow streamlining may be considerably improved. The brainstem/spinal cord Dmax objectives indicate the most conservative gains from workflow streamlining where removing on-unit measurements resulted in 13 more false positive replan indications for the full study cohort over 35 months. However, on-unit image registration and measurements for the cohort are estimated to take 275 person-hours total (approximately 2 minutes/patient), significantly longer than re-CT and dose recalculation for the 13 false positive cases.

The simplified criteria for dosimetric objectives contain anatomically unrelated OARs, indicating correlations with plan quality, where the proximity of target volumes to OAR may have increased OAR doses. In keeping with general treatment planning principles, healthy tissues doses likely were distributed among multiple OAR in an attempt to meet treatment planning criteria. For example, patients appear to be at risk of increased parotid gland dose given high initial parotid gland doses as well as high planned brainstem and spinal cord doses. The “AND” format of the simple patient selection guidelines is well-suited to capture these complex effects and reflects the underlying nature of RF algorithms.

In practice, we expect that the simple patient selection guidelines will be most efficiently implemented using basic treatment planning system scripting capabilities, and ultimately, that patient data may be continuously incorporated into RF model development *via* an auxiliary workflow. However, the simple guidelines are amenable to be pinned to a dosimetrist or booking clerk’s wall for reference. The RF models and simplified guidelines were developed specifically for our institution’s cohort and treatment practices; application to other practices must be carefully reviewed. For example, our center’s radical (chemo)radiotherapy approach for these patients used two dose levels (high-dose CTV = 70 Gy, low-dose CTV = 59.4 Gy). Although this is a common practice, some centers may treat primary disease, high-risk and low-risk lymphatics with three dose levels, potentially affecting the incidence of OAR dose violations. While not statistically significant, slight improvements of the simplified criteria over full RF models may result from the simpler nature of the criteria (i.e., lower variance), and/or possible improvements in our institutions patient planning, immobilization, and on-unit image guidance. Although we strived to produce a comprehensive set of ART objectives, it is not exhaustive and some objectives, such as losses in CTV coverage, could not be modelled due to DIR workflow errors specific to delineation of this anatomical structure.

A further limitation of this study is the use of last-acquired CBCT images for each patient to characterize during-treatment anatomical changes. This approach was motivated by the high resource costs associated with aggregating data for the study cohort, mainly arising from the manual inputs required for image DIR between planning CT and CBCT images. Assuming that patient anatomy was like the last-acquired CBCT for all images overestimates the clinical benefit of ART. However, as our focus is patient selection for ART, the greater “signal” of these images has been used to increase the ability of models to detect anatomical/dosimetric changes. In addition, this approach allowed us to produce a larger and more diverse patient cohort with the aim of developing robust ART models, as compared to processing multiple images per patient.

The timing of ART replanning is generally recommended during the first three weeks of treatment (3), however, timing may vary by objective. Although replan timing falls beyond the scope of the present study, is the focus of ongoing work.

The study design presented may be used to develop ART patient selection criteria for other sites, such as lung, cervix, and anal canal patients. Selection of patients for ART assessment are expected to vary depending on the number and proximity of OARs, and nature of acute toxicities and random *vs.* systematic interfractional anatomical changes.

## Data Availability Statement

The datasets presented in this article are not readily available because the conditions of ethics approval do not currently allow data sharing at this time. Requests to access the datasets should be directed to Sarah.Weppler@albertahealthservices.ca.

## Ethics Statement

The studies involving human participants were reviewed and approved by the Health Research Ethics Board of Alberta. Written informed consent for participation was not required for this study in accordance with the national legislation and the institutional requirements.

## Author Contributions

Overall study design was jointly proposed by SW, WS, CS, and HQ. HQ and SW coordinated data collection. SW conducted the trend and workflow QA analyses, RF modelling and development of the heuristic. JD, NH, CV, and CB assisted with data collection, with additional processes scripted by LV. SW prepared the initial manuscript with WS, CS, and HQ. All authors contributed to the article and approved the submitted version.

## Funding

This work was supported in part by the Natural Sciences and Engineering Research Council of Canada – Canada Graduate Scholarship (CGS-D) to SW.

## Conflict of Interest

The authors declare that the research was conducted in the absence of any commercial or financial relationships that could be construed as a potential conflict of interest.
