# Supplementary material for: Determining Clinical Patient Selection Guidelines for Head and Neck Adaptive Radiation Therapy Using Random Forest Modelling and a Novel Simplification Heuristic
Source: Front Oncol. 2021 Jun 7;11:650335. doi: 10.3389/fonc.2021.650335 (PMC8216638; doi:10.3389/fonc.2021.650335)
Supplement: Supplementary file 1 [file DataSheet_1.docx]

Supplementary Material

# Retrospective CT-CBCT Measurements

Figure S.1: Summary of retrospective measurements acquired on rigid alignments of planning CT and end-of-treatment on-unit CBCT images

*Shrinkage of CBCT external contour relative to CT external contour is indicated by a negative value.

| ***Description:*** | ***Reference Location:*** | ***Measurement:*** |
| --- | --- | --- |
| $\boldsymbol{\Delta}$**Face diameter:** For axial slices inferior to the maxilla and superior to the mandible body, the maximum difference in lateral face diameter (across the oropharynx).* | 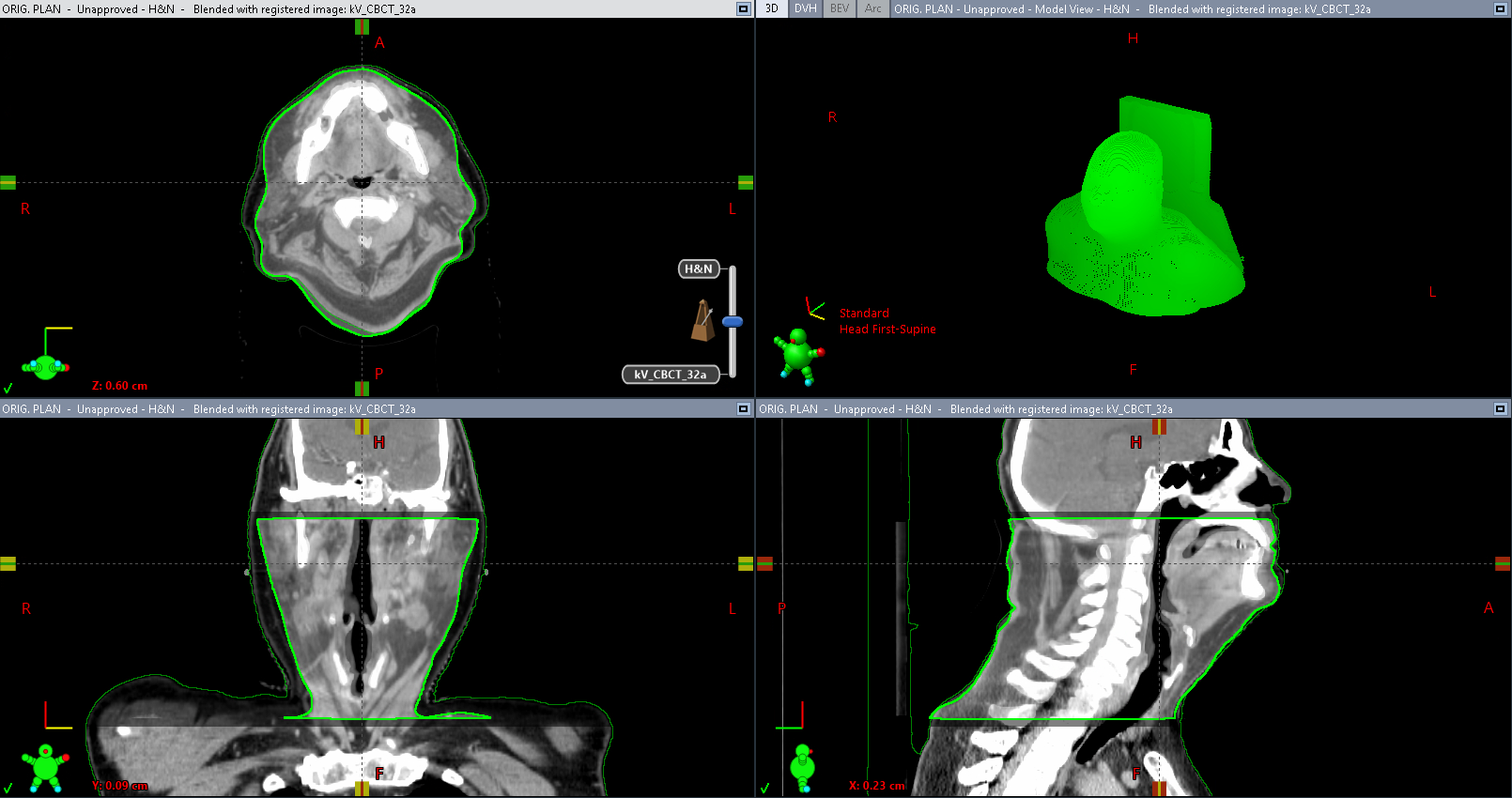 | 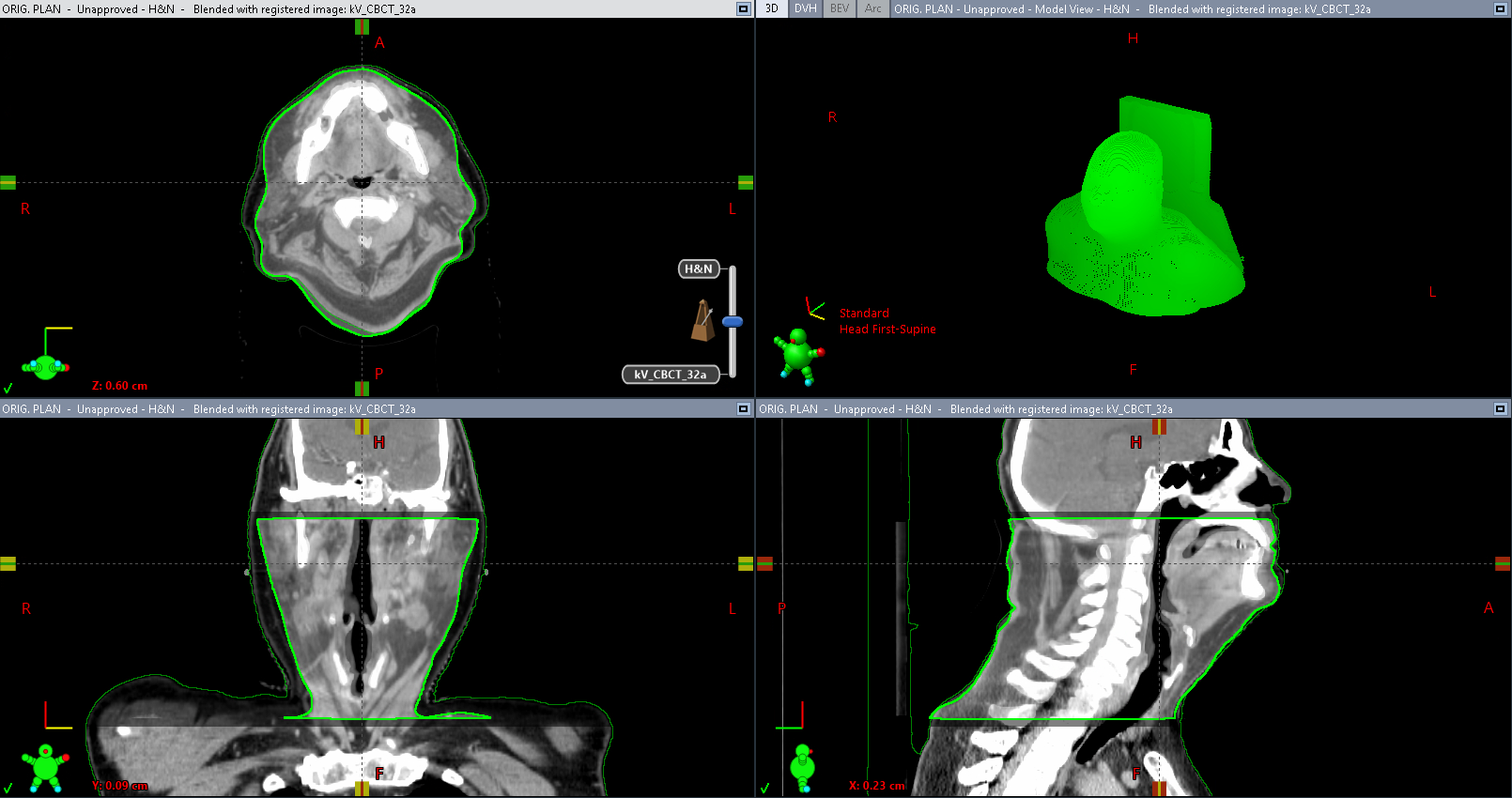 |
| $\boldsymbol{\Delta}$**Neck diameter:** For the axial slice inferior to the hyoid, the maximum difference in lateral neck diameter anterior to the vertebral body.* | 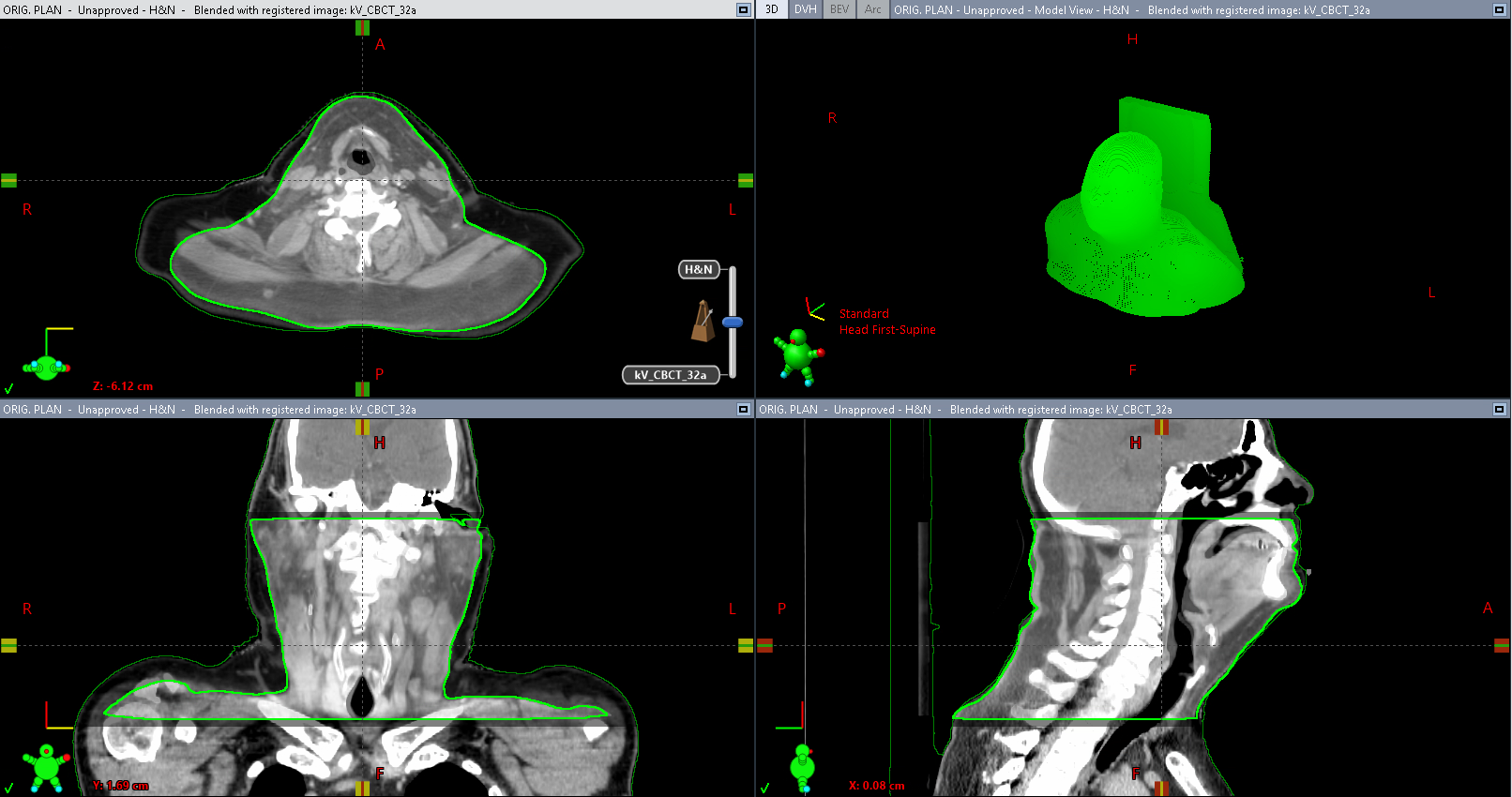 | 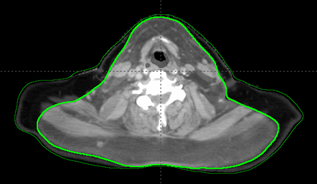 |
| $\boldsymbol{\Delta}$**Neck/shoulder contour:** With the coronal slice posterior the acromio-clavicular joint, the maximum difference in external contour below ear lobes (of right and left sides).* | 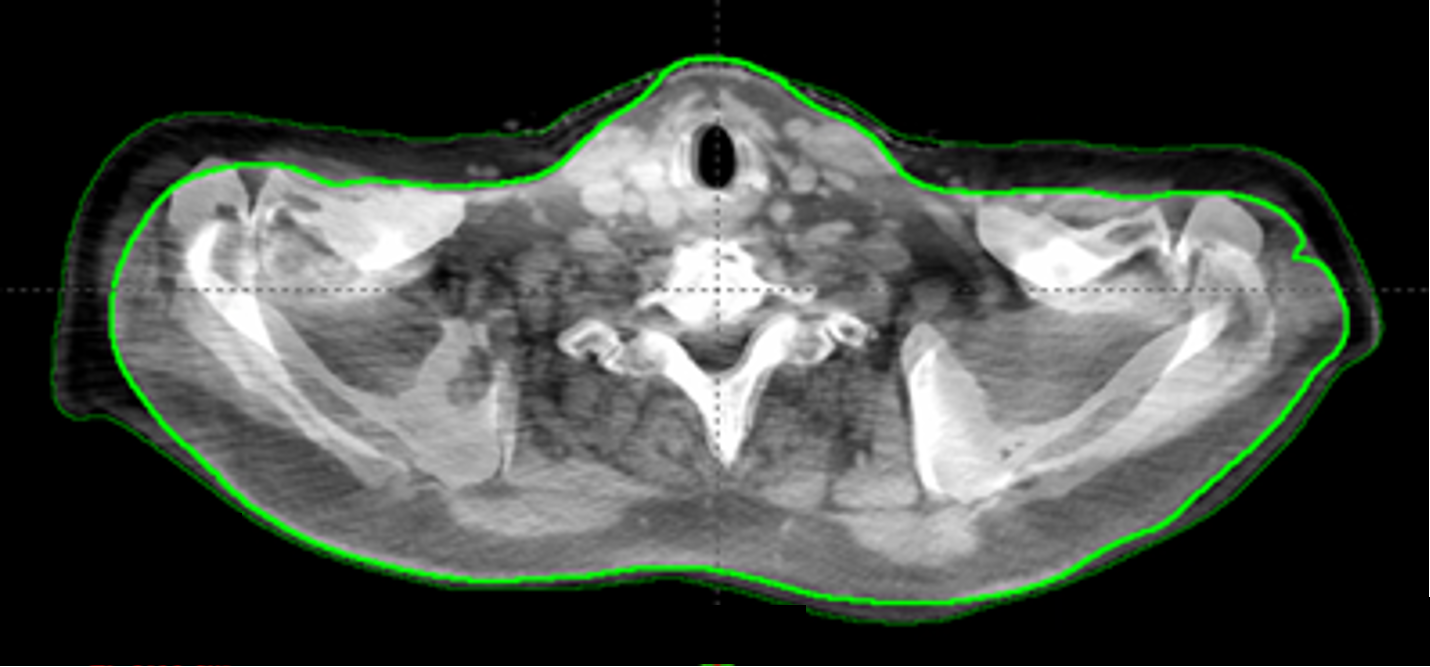 | 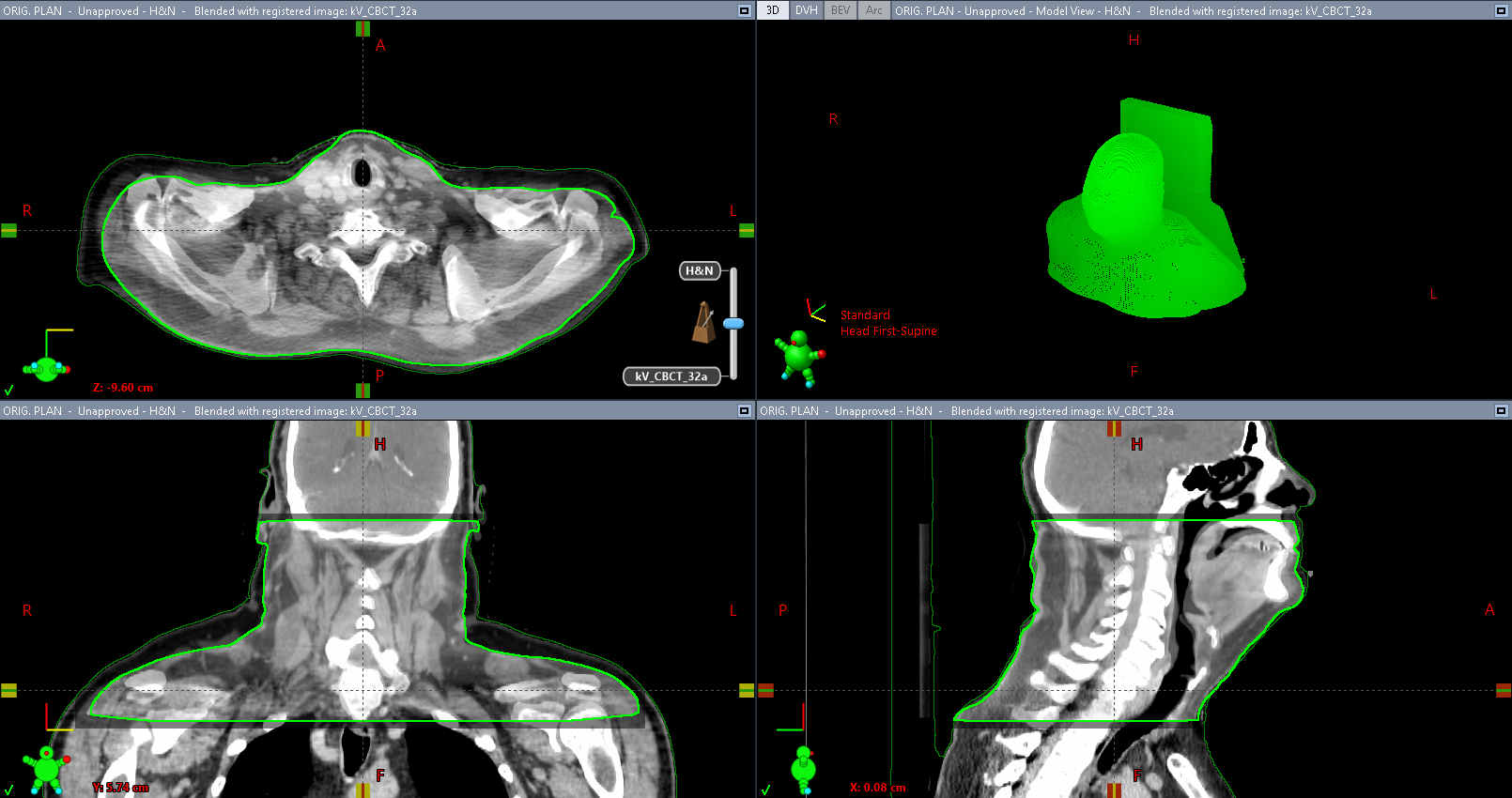 |
| $\boldsymbol{\Delta}$**Head position:** On the axial slice inferior to the C1 vertebral foramen, the difference in mandible position. Posterior shift is indicated by a negative value. | 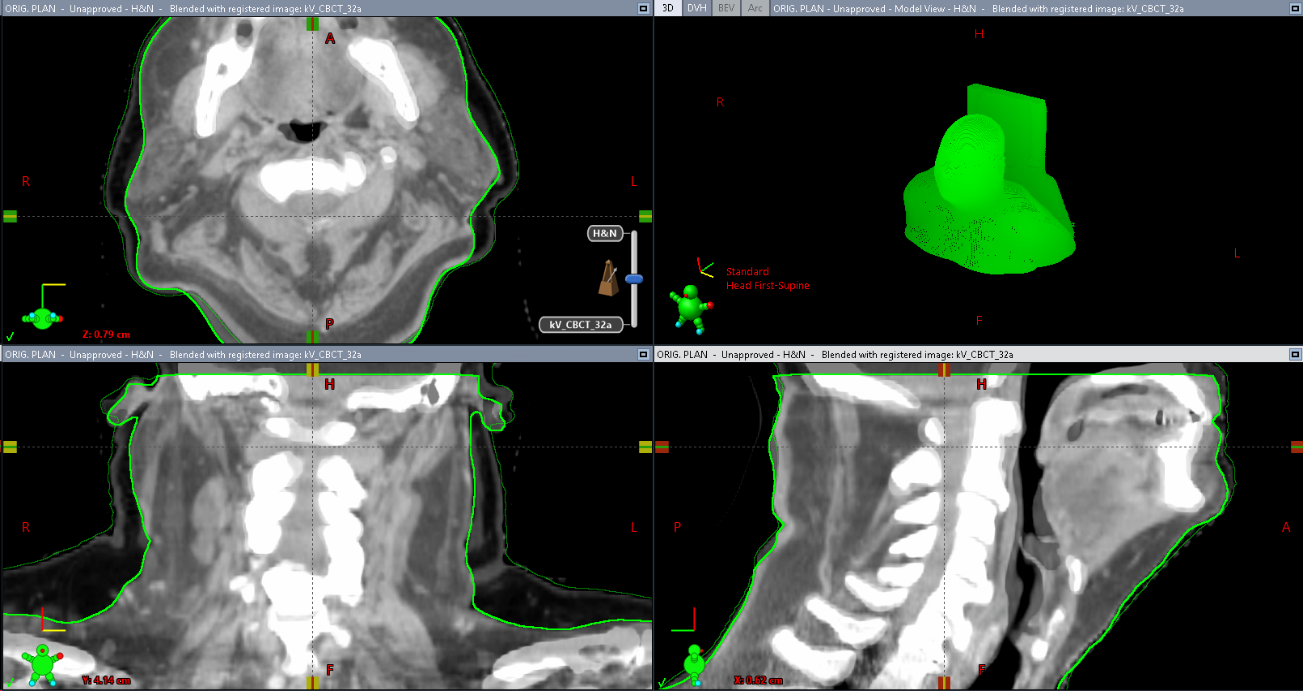 | 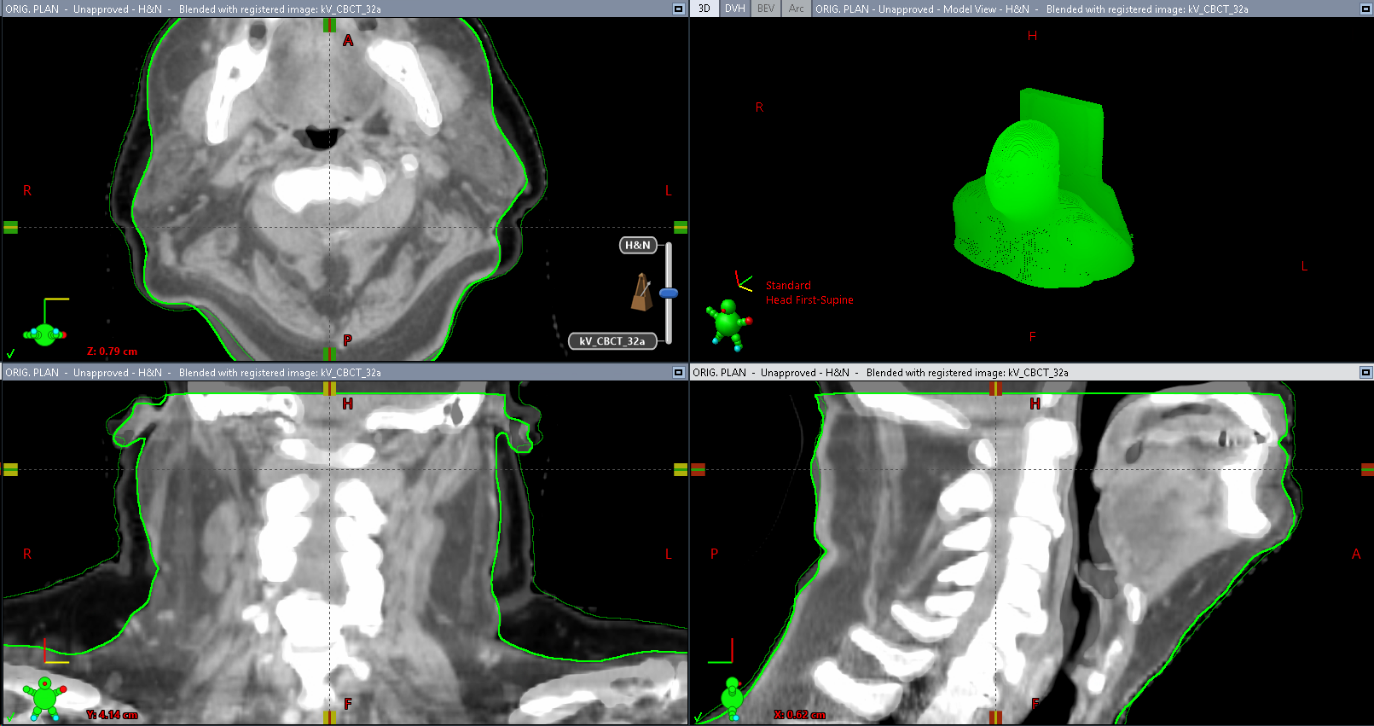 |
| $\boldsymbol{\Delta}$**Chin position:** For the sagittal slice at midline, the difference in position of the mandible body. Inferior shift is indicated by a negative value. | 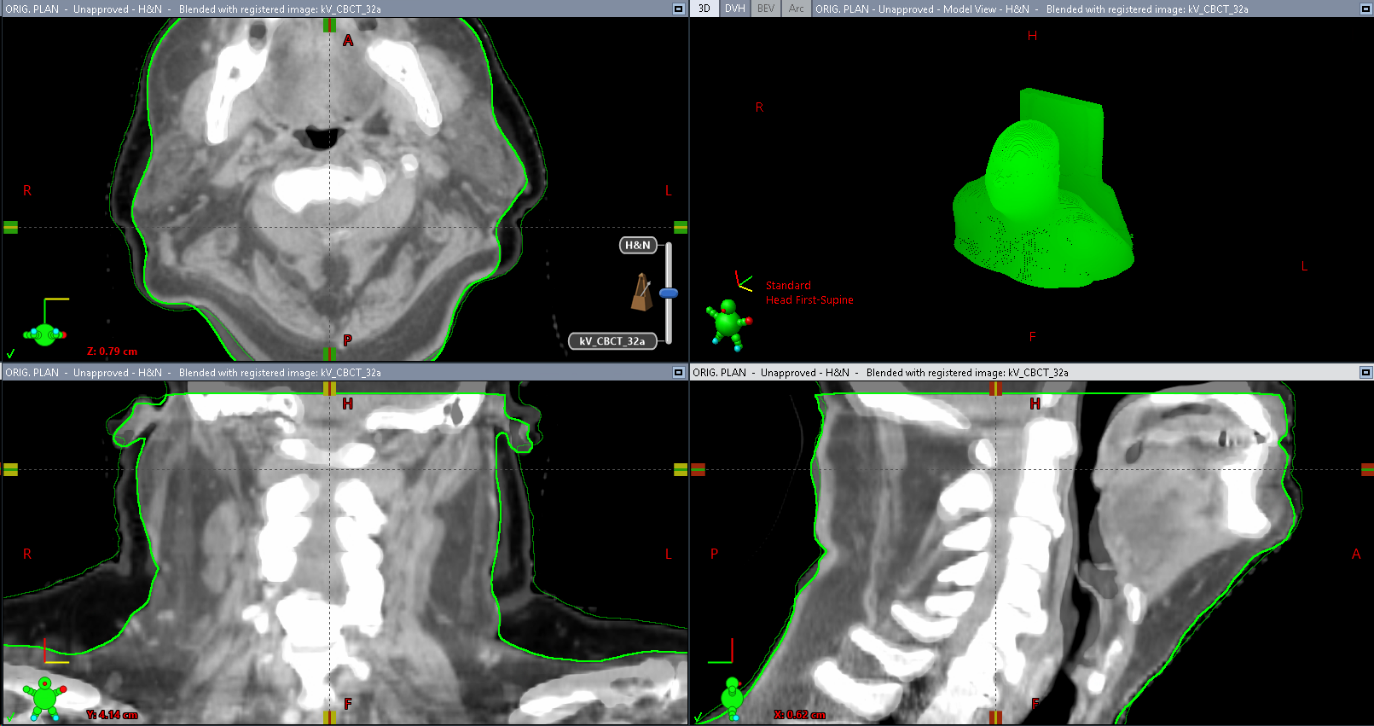 | 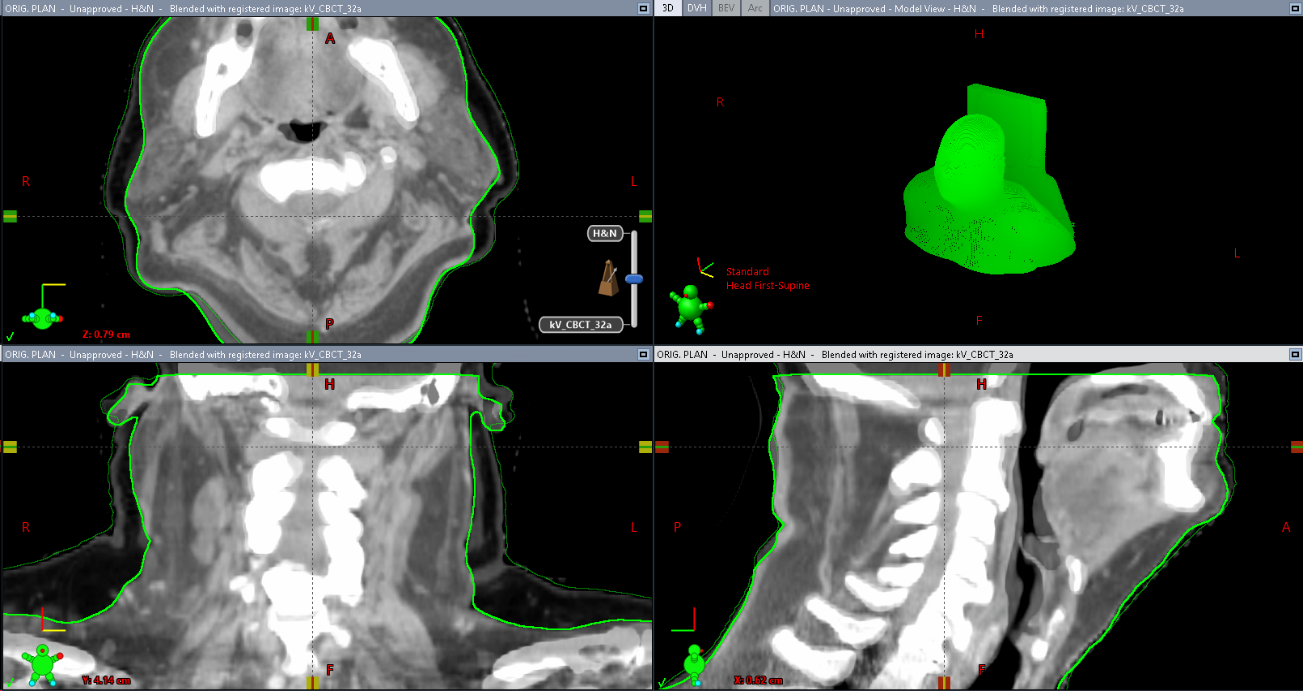 |

Figure S.1 (Continued): Summary of retrospective measurements acquired on rigid alignments of planning CT and end-of-treatment on-unit CBCT images

# Deformable Image Registration Quality Assurance

## Quality Assurance Test Case Selection

*Shrinkage of CBCT external contour relative to CT external contour is indicated by a negative value.

| ***Description:*** | ***Reference Location:*** | ***Measurement:*** |
| --- | --- | --- |
| $\boldsymbol{\Delta}$**Shoulder position:** Two measurements per side with respect to the position of the acromioclavicular joint:  1. Difference in acromioclavicular joint on the axial view. (e.g., $x_{L}$, $x_{R}$)  2. Difference in acromioclavicular joint on the coronal view. (e.g., $y_{L}$, $y_{R}$)  Final value is the maximum of $\sqrt{x_{L}^{2}+y_{L}^{2}}$ and $\sqrt{x_{R}^{2}+y_{R}^{2}}$. | 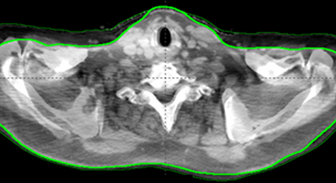 | 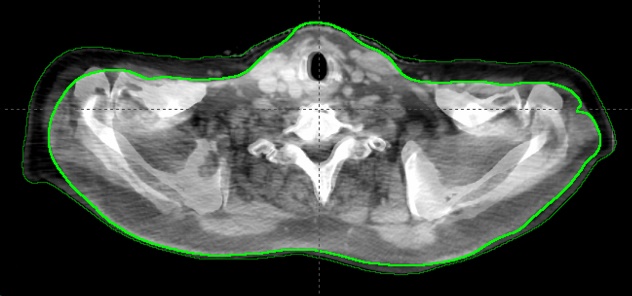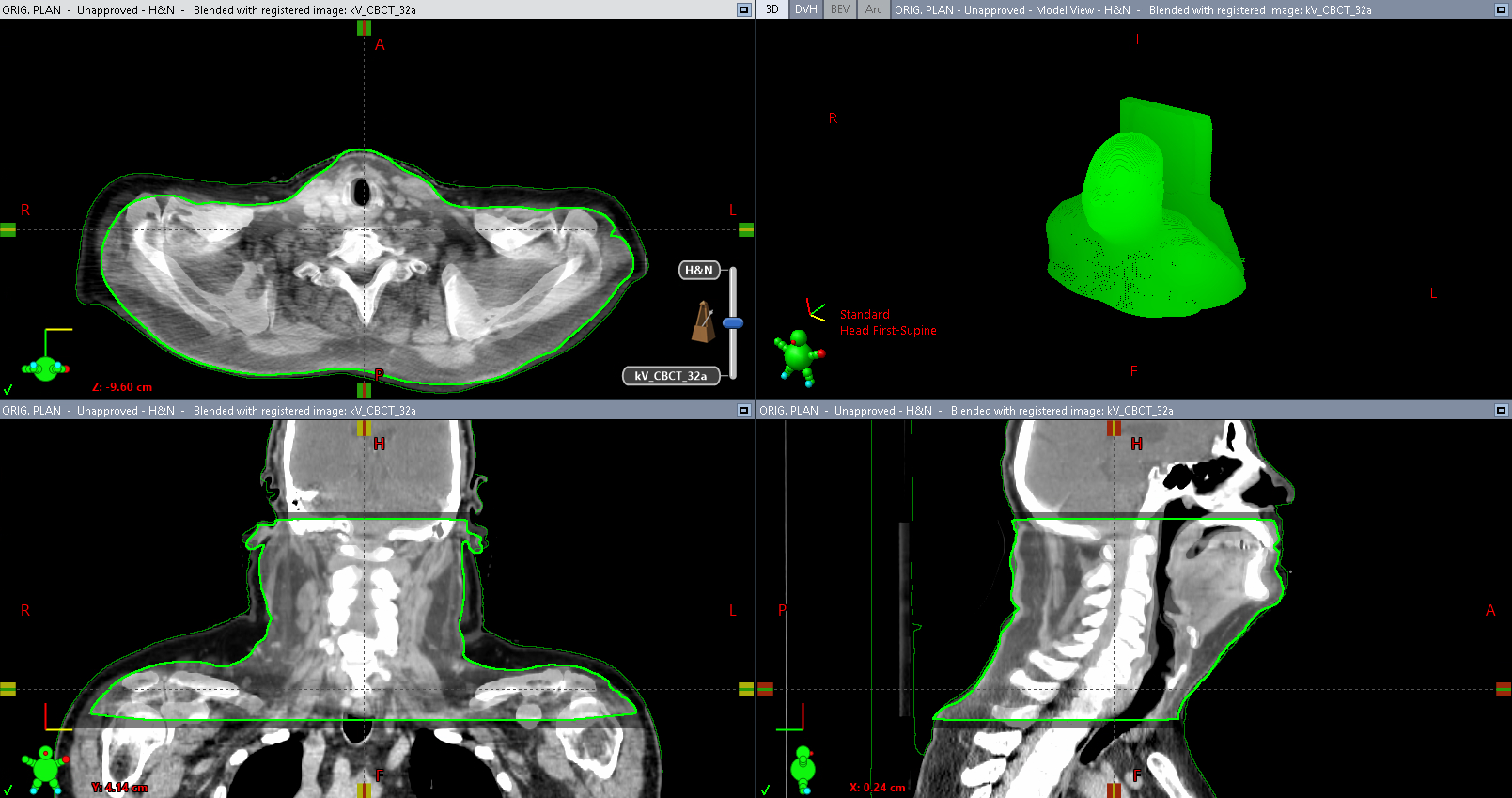 |

Due to its resource-intensive nature, we considered a subset of the 250 synthetic CTs for deformable image registration (DIR) quality assurance (QA). The process used to select cases for QA was motivated by Weppler *et al*. 2020a: k-Medoid data clustering characterized planning CT vs. synthetic CT differences either arising from inter-fractional anatomical changes or DIR error (Figure S.2). Cluster centres (“medoids”) provided 15 candidate QA cases. After removing 6 single-image outlier clusters, we obtained our QA set of 9 images. Projection of the clusters into the first two principal component dimensions indicated that clusters identified systematic differences in synthetic CTs; however, differences were not statistically significant according to Kruskal Wallis tests with Benjamini Hochberg multiple testing corrections (false discovery rate = 5%).

## Geometric Assessment of Deformable Image Registration Output According to the AAPM’s Task Group 132 Report

*Shrinkage of CBCT external contour relative to CT external contour is indicated by a negative value.

To ensure the geometric comparability of DIR and clinician contours, two radiation oncologists specializing in head and neck cancer produced one set of consensus contours for each of the 9 QA patients on the last- acquired CBCT images. We rigidly propagated synthetic CT contours to the CBCTs, trimmed them to the smaller CBCT field of view, and quantified physician vs. DIR Dice similarity index (contour overlap) and mean distance to agreement (contour displacement) according to the AAPM’s Task Group (TG) 132 report (Brock *et al.* 2017).


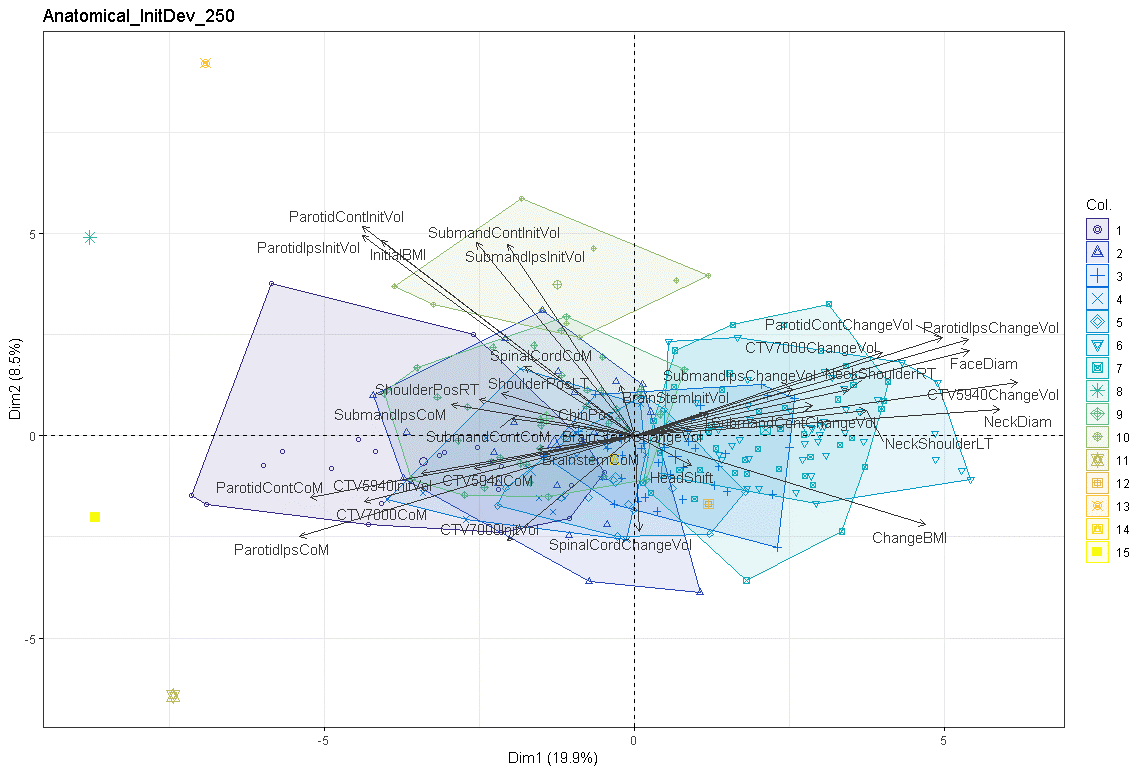


Dimension 1 (19.9% Variance Explained)

Dimension 2 (8.5% Variance Explained)

Figure S.2: Visualization of the data clusters used to select cases for DIR QA. Anatomical differences between patient planning CTs and synthetic CTs are grouped using k-medoid clustering and have been projected into the first two principal component dimensions for visualization. Arrows denote the principal component loadings; the longest arrows indicate which parameters were of most importance in separating clusters. Performing DIR QA on cluster centres helped to ensure that important anatomical changes and relevant DIR error types were included in the QA results.

DIR contours were consistent with the physician contours according to TG-132 recommendations for all structures except submandibular glands. Mean distance to agreement averaged 2.4 mm across the remaining contours (range = 1.6-3.2 mm), less than the maximum CBCT voxel dimension of 2.5 mm. Average Dice similarity index was 0.65 (range = 0.55-0.74). This is comparable to the interobserver variability between physicians when contouring on images with streaking image artifact (Hansen *et al.* 2017), characteristic of many CBCT images. In contrast, average submandibular gland mean distance to agreement was 4.6 mm and Dice similarity index was 0.49; delivered submandibular gland dose was omitted from further analysis.

## Dosimetric Assessment of Deformable Image Registration Output

A recent study in the literature recommends that TG-132 geometric QA assessments be supplemented with a dosimetric analysis (Lim *et al*. 2019). Therefore, we rigidly propagated physician CBCT contours to the corresponding synthetic CTs, trimmed DIR contours to be consistent with the field of view of physician contours, and recalculated dose. Differences between the dose to a physician structure, ${Dose}_{Phys}$, and dose to a DIR structure, ${Dose}_{DIR}$, were averaged across the QA cases (i.e., workflow “noise”). To consider the implications of these discrepancies on our machine learning model development, we divided by the inter-fractional dose changes between the planning CT, ${Dose}_{pCT}$, and CBCT, ${Dose}_{DIR}$ (i.e., workflow “signal”):

$$\frac{{Dose}_{Phys}-{Dose}_{DIR}}{{Dose}_{pCT}-{Dose}_{DIR}}$$

A resulting ratio less than one indicated that discrepancies in estimated dose due to contour variations were small compared to systematic dosimetric changes of interest.

We obtained ratio values of: 0.5 for brainstem/spinal cord, 0.3 for parotid glands, 0.5 for pharyngeal constrictor and 0.2 for high-dose CTV D2%. The ratio value for high-dose CTV D95% was well above one, exacerbated by poor CBCT image quality and variations in target contouring conventions; delivered high-dose CTV target coverage was omitted from further analysis.

# Tolerances for Random vs. Systematic Changes in Estimated Objective Values

Results of the trend analysis are shown in Figure S.3. Due to the large amount of daily noise in the data, a linear fit was used to reduce model variance. Visual review of all patients/structures indicated no other specific trends (e.g., quadratic, exponential).

Trend and quartile tolerances are summarized in Table S.1. For objectives based on two structures, such as increases in dose to brainstem/spinal cord, the larger standard deviation was used to define the deviation tolerance. Variations in patient setup time appeared to be random with progression through treatment and this objective was omitted from further analysis. In contrast to the deformable image registration analysis, we randomly selected patients for this sub-analysis as the nature of during-treatment trends was unknown.


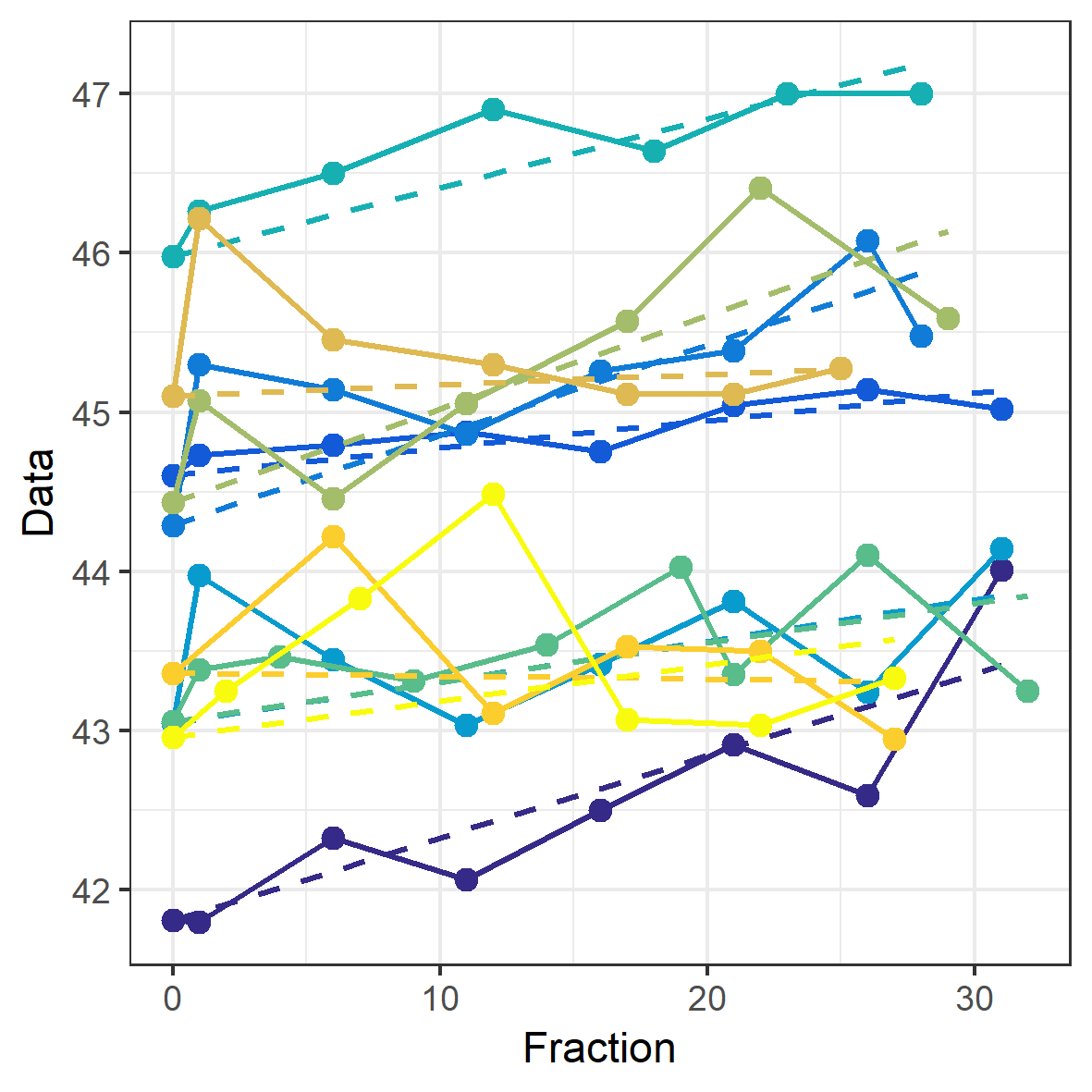

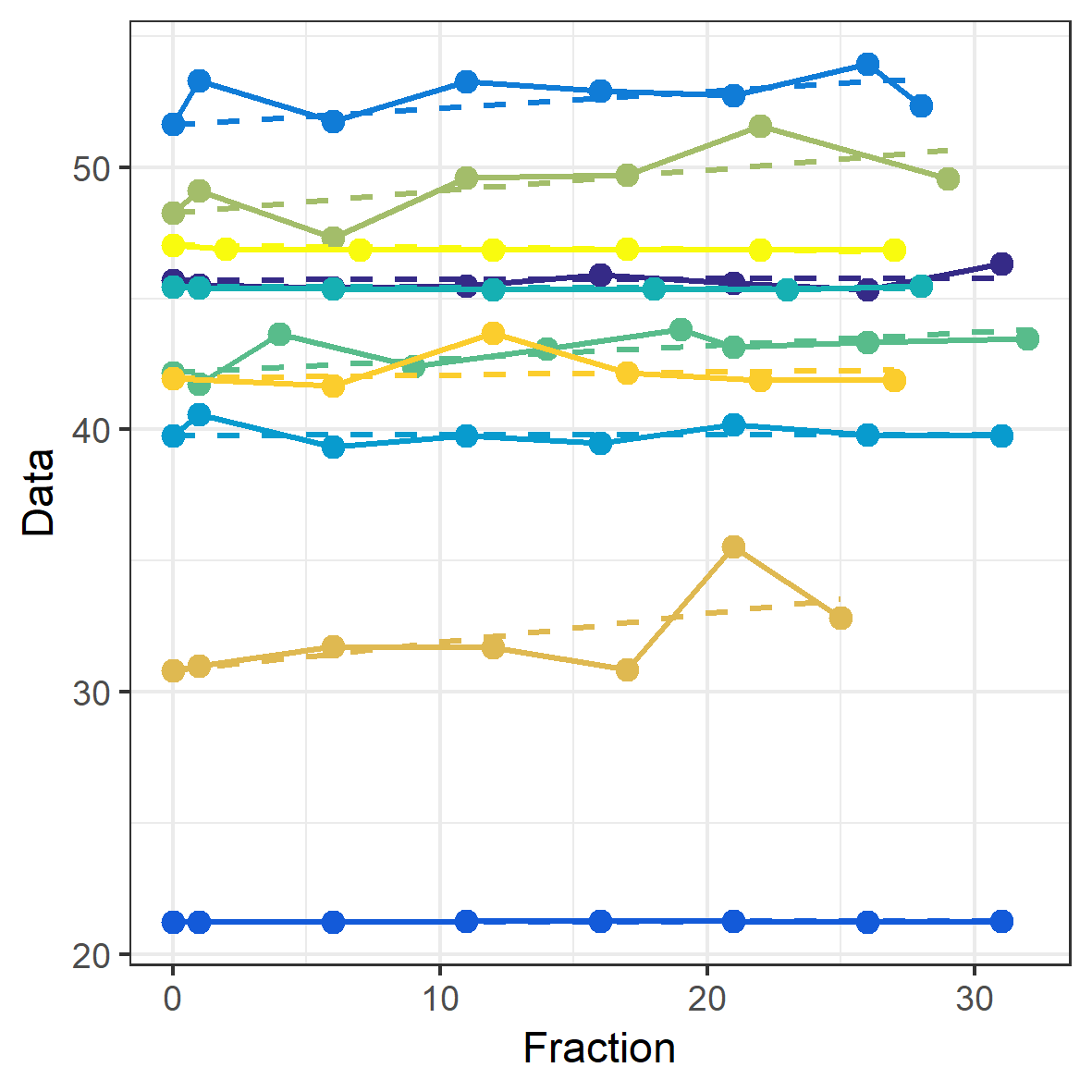


Dose (Gy)

Fraction Number

Fraction Number

Dose (Gy)

**i. Brainstem D0.03cc**

**ii. Spinal cord D0.03cc**

Figure S.3: Inter-fractional changes in dosimetric, clinical, and volumetric parameters. Solid lines denote patient-specific trends and dashed lines the corresponding linear fit.


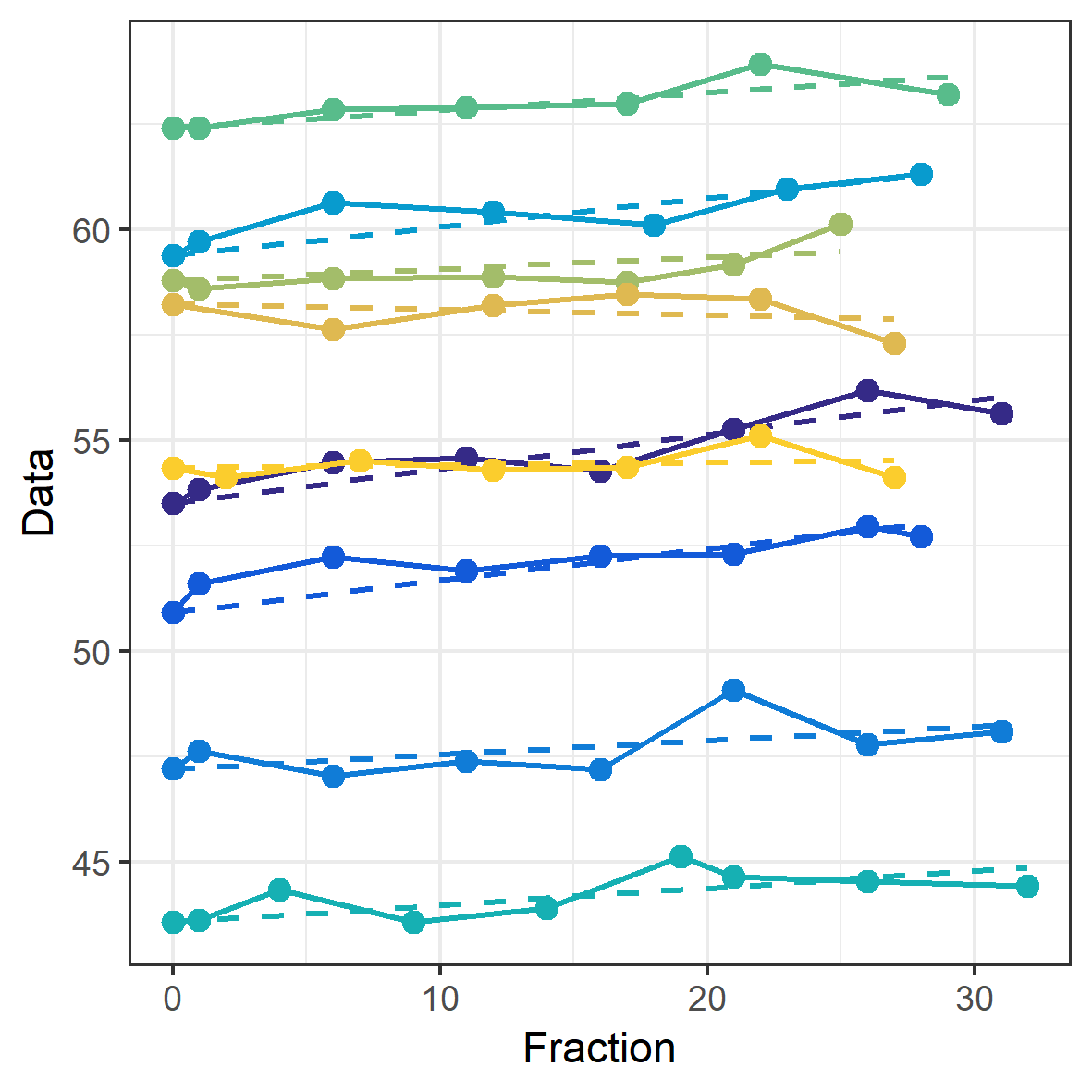

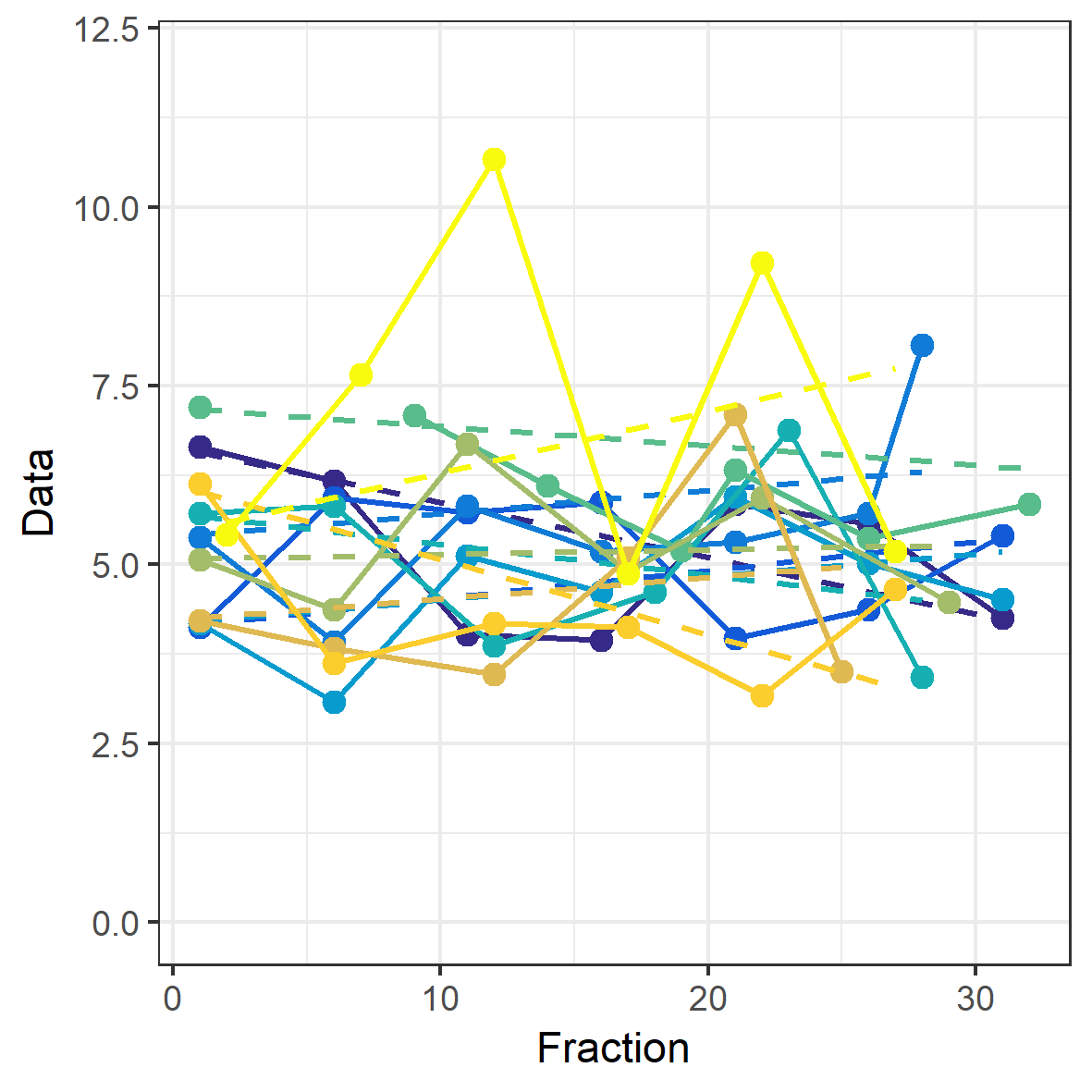

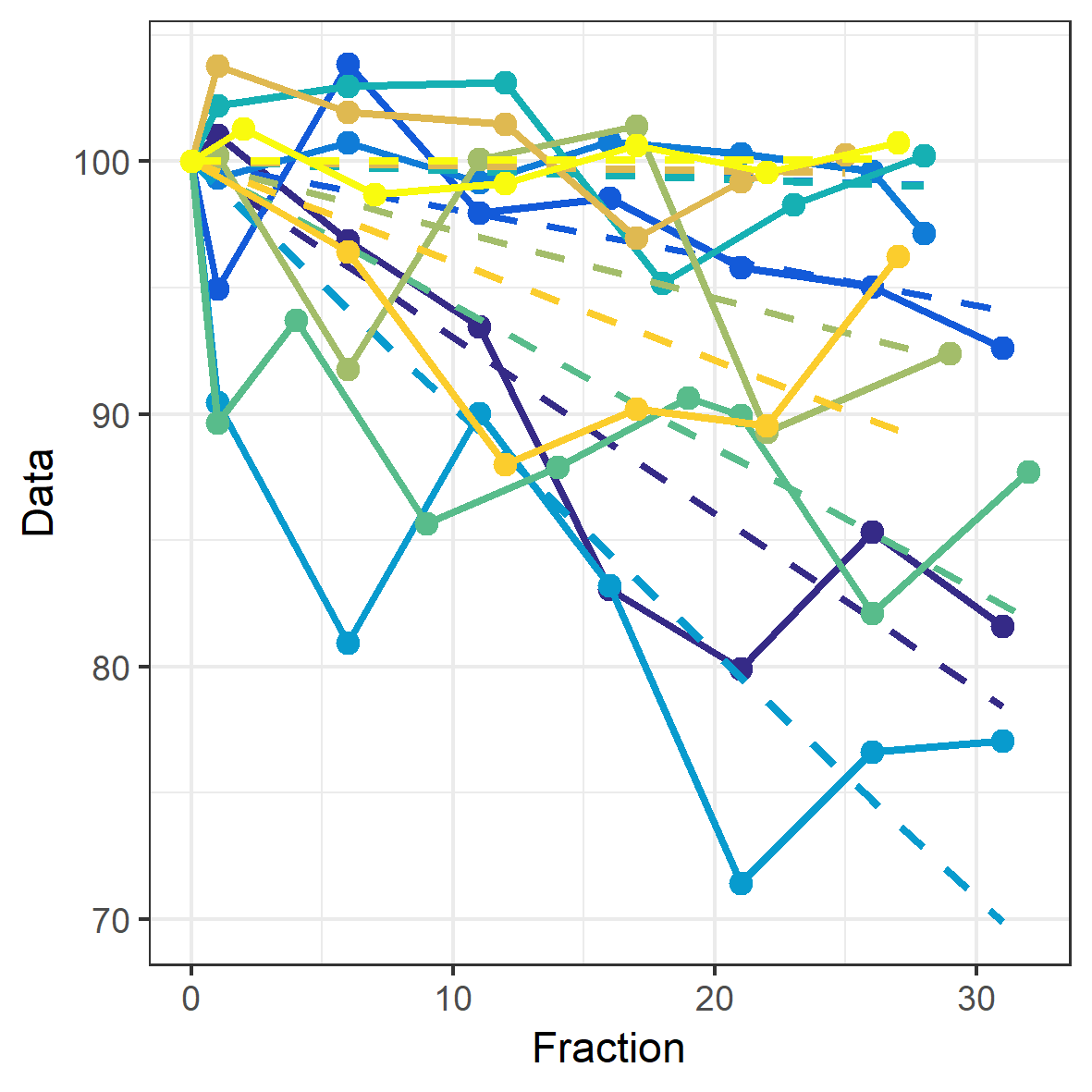

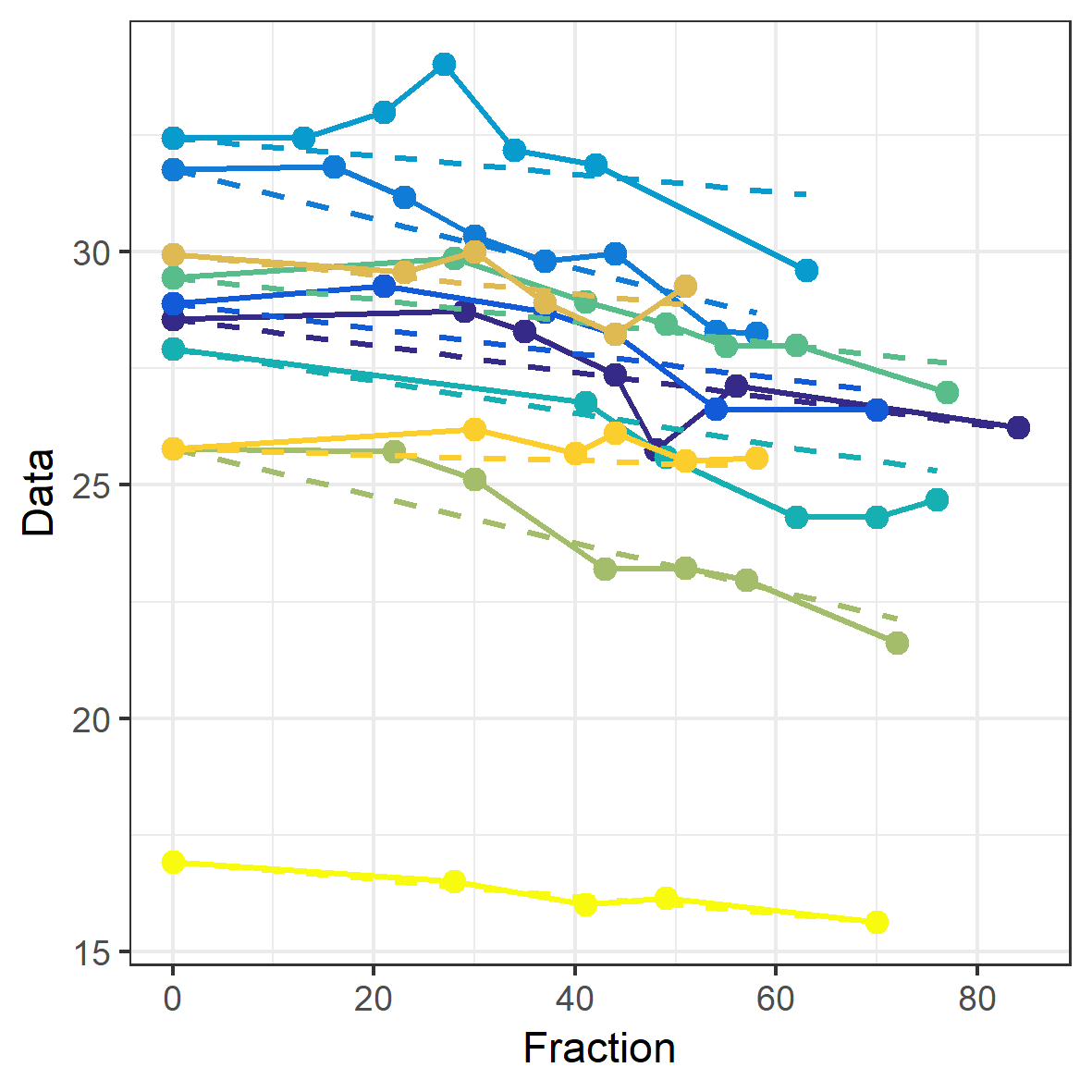


**v. Pharyngeal constrictor Dmean**

**vi. BMI**

**vii. High-dose CTV volume**

**vii. Setup time**

Days Since Treatment Start

Fraction Number

Fraction Number

Fraction Number

Dose (Gy)

BMI

Relative Volume (%)

Time (minutes)

*****

*****


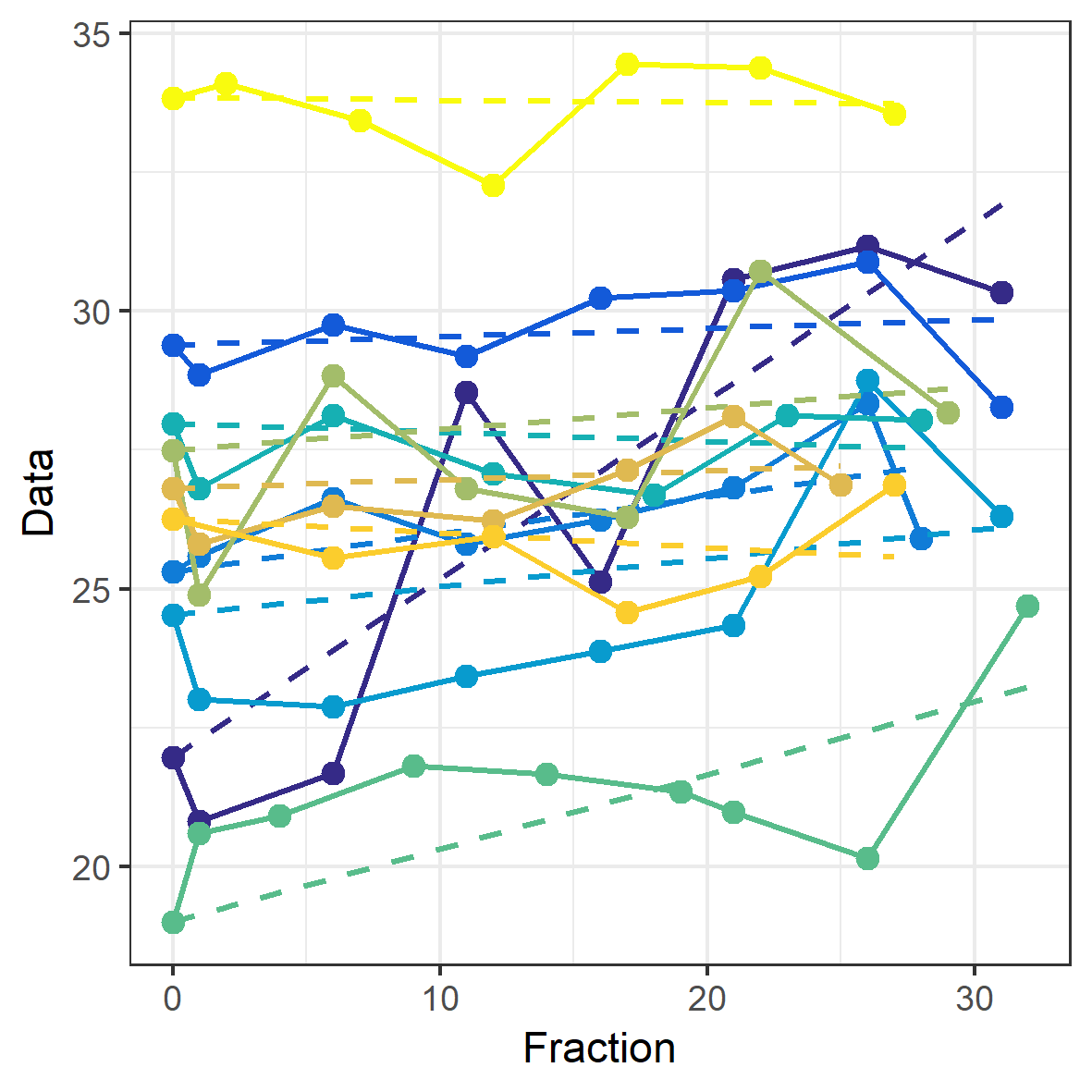

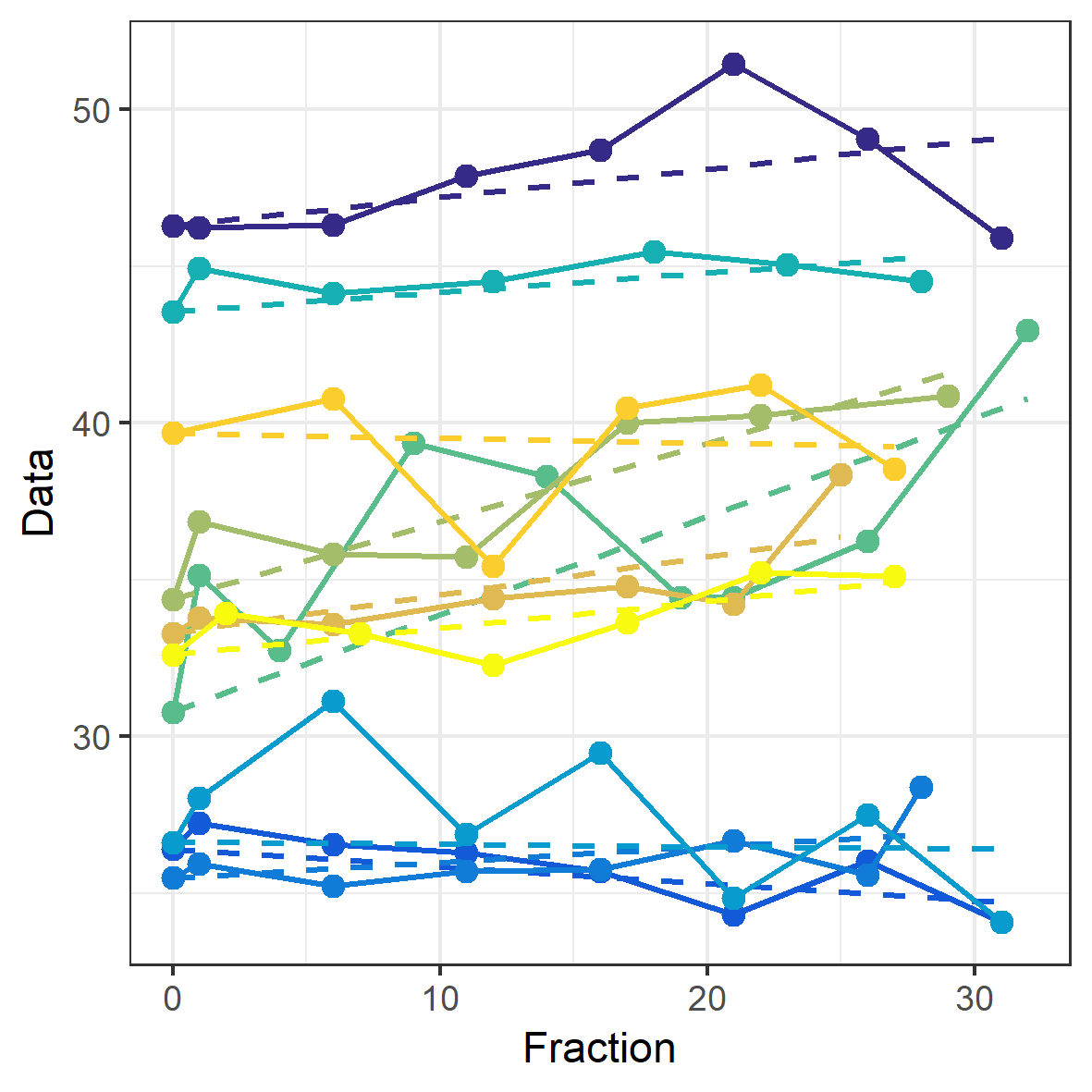


**iii. Ips. parotid gland Dmean**

**iv. Cont. parotid gland Dmean**

Fraction Number

Fraction Number

Dose (Gy)

Dose (Gy)

Figure S.3 (Continued). *Outlier point has been trimmed from these graphs at the indicated fraction to improve visualization.

Table S.1: Summary of objectives and standard deviation and quartile tolerances

| ***ART Objective*** | ***Trend analysis random error deviation tolerance (% of patients with violation)*** | ***Tolerance isolating the quartile of patients with the most unfavourable changes*** |
| --- | --- | --- |
| 1. Increase in brainstem/spinal cord Dmax | 1.0 Gy (20%) | 0.8 Gy |
| 1. Increase in parotid gland Dmean to the spared gland | 2.2 Gy (27%) | 0.9 Gy |
| 1. Increase in pharyngeal constrictor Dmean | 0.8 Gy (47%) | 1.5 Gy |
| 1. Increase in submandibular gland Dmean to the spared gland | (Omitted due to geometric discrepancy between DIR and physician submandibular gland contours.) | |
| 1. Decrease in high-dose CTV D95% target coverage | (Omitted due to dosimetric discrepancy between DIR and physician high-dose CTV contours.) | |
| 1. Increase in high-dose CTV D2% target hotspot | (Omitted as too few patients exceeded the planning objective to produce a predictive model.) | |
| 1. Increases in high-dose CTV volume | 6.7% (8.0%) | 1.0% |
| 1. Decreases in patient BMI | 1.8 kg/m^2^ (68%) | 3.4 kg/m^2^ |
| 1. Increases in on-unit patient setup time from the first kV-orthogonal image to beam-on | (Omitted as variations appeared random with time.) | |

# Random Forest Model Development

We considered various combinations of normal/violation formats, input parameters, and random initialization seeds during random forest (RF) model development:

- Objective formats: PC_CI; PC_Q; ALARA_CI; ALARA_Q (see Figure S.4). We applied all 4 formats to the ART objectives with treatment planning objectives (brainstem/spinal cord Dmax, parotid gland Dmean, and pharyngeal constrictor Dmean). Only the ALARA_Q format was applied to the remaining objectives (decreases in patient BMI, increases in high-dose CTV volume).
- Subsets of input predictors (15 total): EMR; pCT; RTx; Obs; EMR/pCT; EMR/RTx; EMR/Obs; pCT/RTx; pCT/Obs; RTx/Obs; EMR/pCT/RTx; EMR/pCT/Obs; EMR/RTx/Obs; pCT/RTx/Obs; EMR/pCT/RTx/Obs.
- Random Initialization (5 total): Seed values of 1 through 5.

In total, this produced: (4$\times$3 $+$ 2$\times$2)$\times$15$\times$5 = 1200 combinations of model parameters.

To produce a receiver operating characteristic (ROC) curve for each set of model parameters, we partitioned the range of objective values into 500 equally-spaced “thresholds”. During five-fold cross validation, we developed a RF regression model on 4 folds and predicted numerical response data on the remaining fold. Prediction values less than a given threshold were classified as “normal” and the remainder as “violation”. Averaging model sensitivity and specificity for each threshold among the five hold-out folds produced a single point on an ROC curve.

Selection of the most effective combinations of objective formats and input parameters followed a Greedy stepwise approach based on Youden index. This process is demonstrated in Figure S.5. We assessed how well a parameter set was able to stratify model performance using the strength of Kruskal-Wallis test significance and visual inspection.


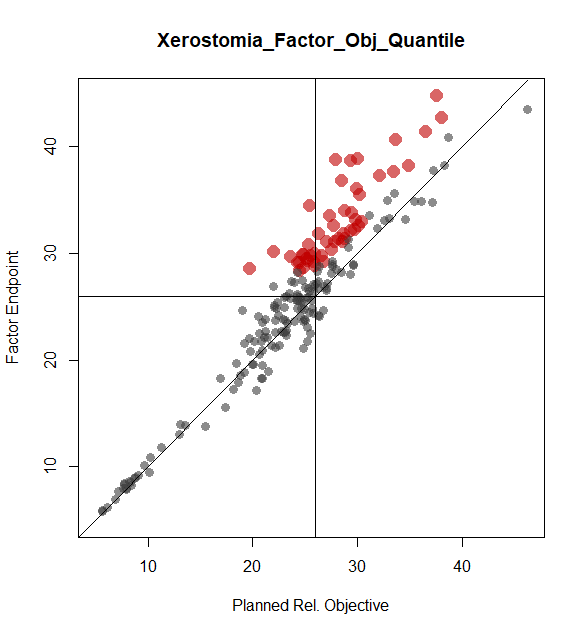

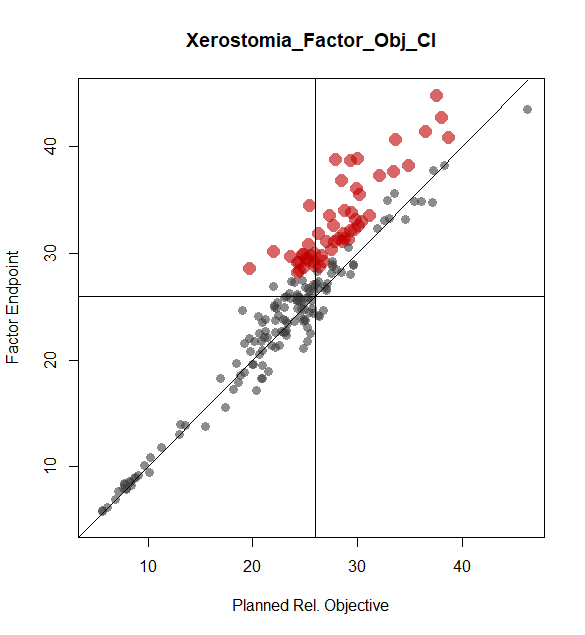

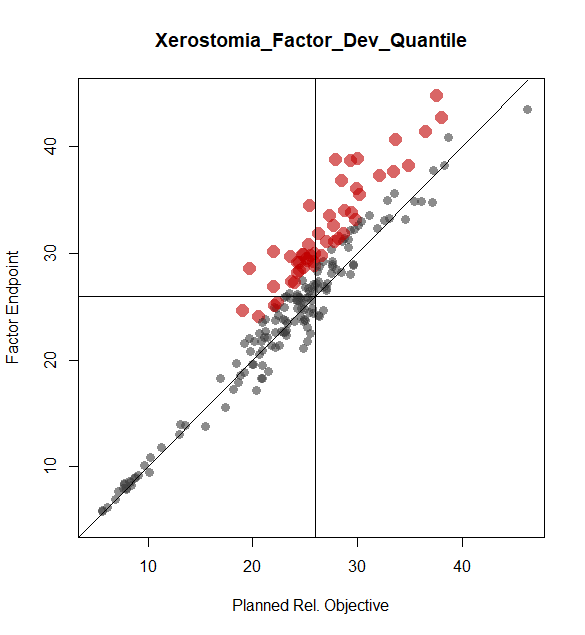

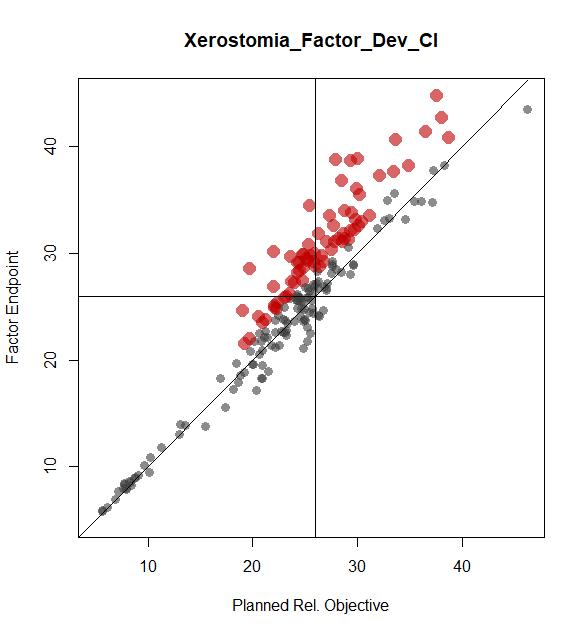


Planned Dose (Gy)

Planned Dose (Gy)

Planned Dose (Gy)

Planned Dose (Gy)

Delivered Dose (Gy)

Delivered Dose (Gy)

Delivered Dose (Gy)

Delivered Dose (Gy)

**i.**

**ii.**

**iii.**

**iv.**

Legend: Violation Normal

Figure S.4: Formatting of the objectives according to planning criteria violations (PC) exceeding 26 Gy and the ALARA principle as demonstrated for planned vs. delivered parotid gland dose. Within each paradigm, we identified the subgroup of patients likely to have systematic inter-fractional changes exceeding random error deviation tolerance derived from the trend analysis (CI), as well as the quartile of patients with the greatest delivered deviations (Q). i. PC_CI, ii. PC_Q, iii. ALARA_CI, iv. ALARA_Q. Numerical response data is the vertical distance of points from the decision boundary (bold line) minus the deviation or quartile tolerance (2.2 Gy for PC_CI and ALARA_CI; 0.9 for PC_Q and ALARA_Q).


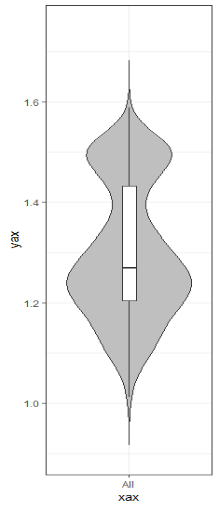

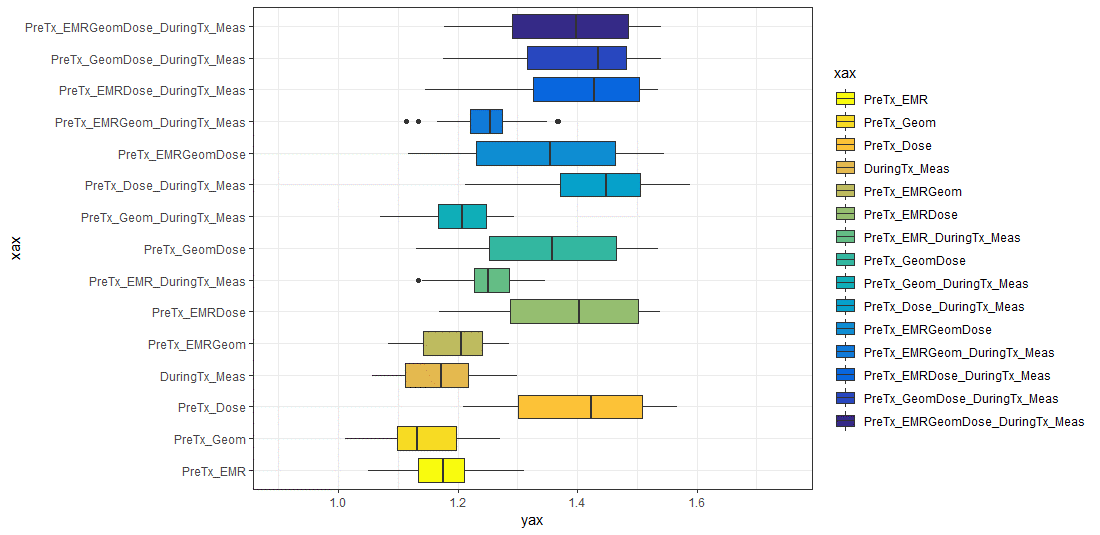

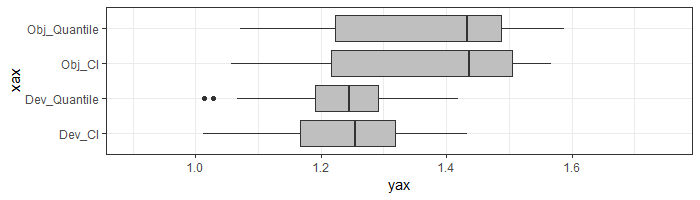

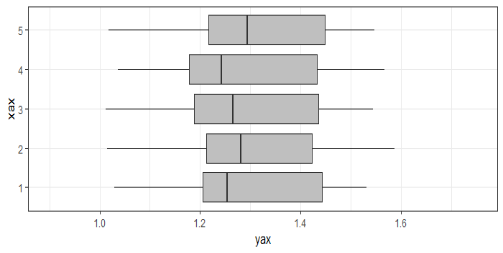


pCT

RTx

Obs

EMR/pCT

pCT/RTx

EMR/RTx

EMR/Obs

pCT/Obs

EMR/RTx/Obs

EMR/pCT/Obs

RTx/Obs

EMR/pCT/RTx

pCT/RTx/Obs

EMR/pCT/RTx/Obs

EMR

ALARA_CI

ALARA_Q

PC_CI

PC_Q

1

2

3

4

5

1.0

1.2

1.4

1.6

**Youden Index**

**Predictor Set**

**Objective**

**Format**

**Random**

**Seed**

All Models


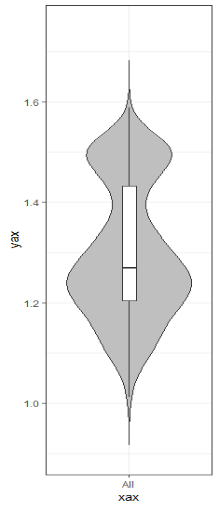

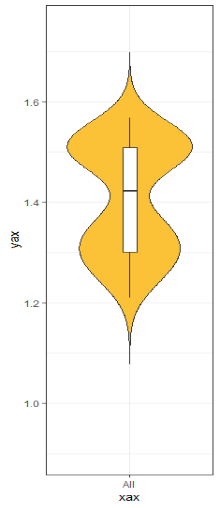

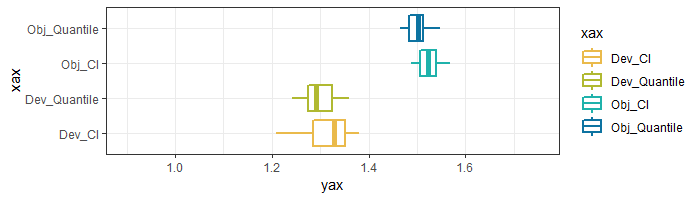

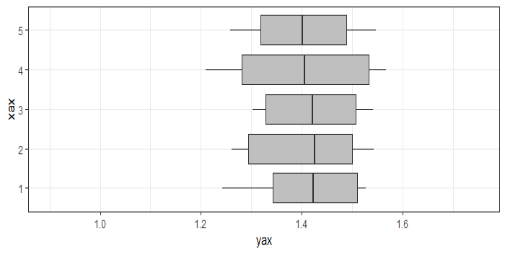


ALARA_CI

ALARA_Q

PC_CI

PC_Q

1

2

3

4

5

1.0

1.2

1.4

1.6

All Models

RTx

**Youden Index**

**Objective**

**Format**

**Random**

**Seed**

Figure S.5: The Greedy stepwise approach used to determine the set of parameters associated with the best-performing models as demonstrated for the parotid gland objective. Top: given all sets of parameters (predictor set,


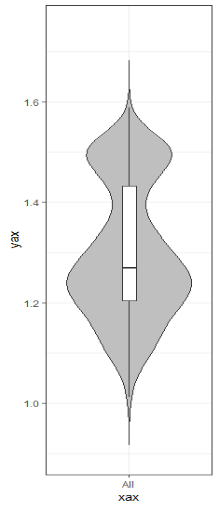

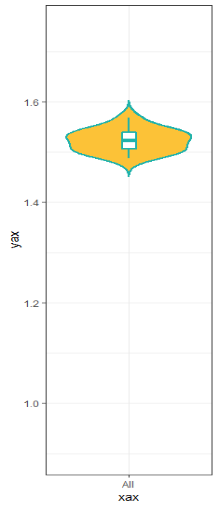

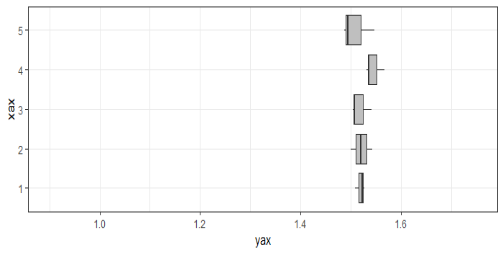


1

2

3

4

5

1.0

1.2

1.4

1.6

All Models

RTx/PC_CI

**Youden Index**

**Random**

**Seed**

normal/violation format, and random initialization), stratifying the performance of models according to input predictor sets best distinguished strong vs. weak performance. Middle: given the most parsimonious predictor set associated with best model performance (RTx), normal/violation format further stratified strong vs. weak results. Bottom: RTx/PC_CI models had the greatest Youden index. No statistically significant differences in Youden index resulting from random initialization seed occurred in any Greedy step, as expected (Kruskal-Wallis statistical tests).

# Heuristic to Develop Simplified Patient Selection Guidelines

We heuristically simplified the best-performing random forest models for each ART objective. This approach, motivated by the heuristic proposed by Weppler *et al*. 2020b allowed us to derive intuitive and easily implementable patient selection guidelines. We demonstrate this process for the model predicting increases in parotid gland dose (formatting: OBJ_CI, predictor set: RTx) (Figures S.6-S.8).

## Demonstrating the Heuristic Process

Inputs for the heuristic included a set of predictors, random forest model sensitivity and specificity across the five random initializations, and corresponding threshold maximizing Youden index from the receiver operating characteristic (ROC) curve analysis. We first ranked predictors in order of decreasing importance, averaged over the five random starts (Figure S.6).

We then determined the common values of high-importance variables occurring at the borderline of “normal” vs. “violation” model predictions. While model performance had previously been estimated using five-fold cross validation (developed on 160 patients from the training set), we generated a new machine learning model on all training data (200 patients). Thresholds converting regression results to categorical normal vs. violation predictions from the cross-validated models were averaged and applied to the new model (e.g., -2.2 Gy for the above model predicting increases in parotid gland dose).

Planned cont. parotid gland Dmean

Planned cont. submand. gland Dmean

Planned pharyngeal constrictor Dmean

Planned ips. parotid gland Dmean

Planned spinal cord D0.03cc

Planned brainstem D0.03cc

Planned low-dose CTV D20%

Planned ips. submand. gland Dmean

Planned high-dose CTV D95%

Planned low-dose CTV D95%

Planned high-dose CTV D2%


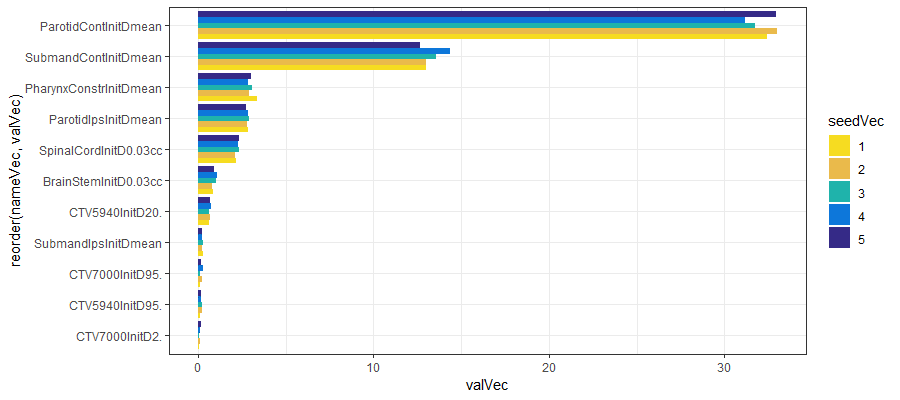


**Variable Importance**

**Input Predictors**

Figure S.6: Importance of input predictors in the random forest model developed to predict increases in delivered parotid gland dose. We input only RTx parameters into the reference model. Random error deviation tolerances were applied to the parotid gland planning constraint of 26 Gy to classify dose increases in the dataset as normal or violation.

For each patient in the training dataset, we substituted observed combinations of high-importance predictor values from the remaining patients in the training cohort. Each resulting simulated data point was input into the new model and labelled according to its normal or violation prediction. For a given high-importance variable, we considered the distribution of values corresponding to violation predictions (Figure S.7). Assessing all substitutions for a given patient, we documented a conservative variable value leading to violations (e.g., 2^nd^ percentile for continuous variables, mode for factor variables). Next, considering these values among all patients, we extracted median values (mode for factor variables) as candidate ART inclusion/exclusion cutoff values in the simplified patient selection criteria.

Given the inclusion/exclusion cutoff values for each variable, we determined the number of variables to retain in the simple patient selection criteria to balance performance and practicality. We determined the sensitivity and specificity of including only the most important variable and corresponding cutoff value; the two highest-importance variables; and so on to a practical limit of 10 variables. To select from these results, we identified which numbers of variables, $v$, produced sensitivity ($sens$) comparable to specificity ($spec$): $\left| {sens}_{v}- {spec}_{v} \right|\leq\min_{v=1,\ldots,10} |{sens}_{v}-{spec}_{v}|+ 0.1$. Of these, we picked the number of variables maximizing Youden index. If Youden index exhibited negligible decreases (i.e., <1%) with the removal of a variable, the simpler criteria was selected, see Figure S.8.


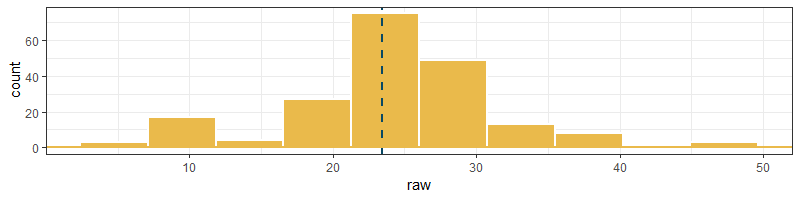


Planned Contralateral Parotid Gland Dmean

Number of Patients


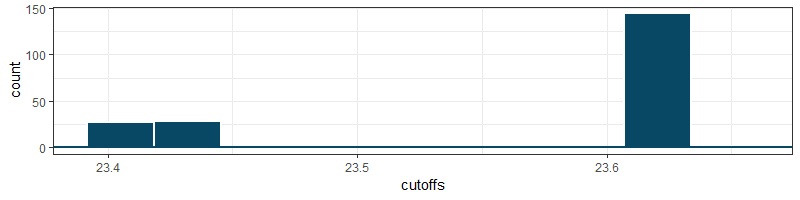


Contralateral Parotid Gland Dmean Candidate Cutoff Values Across the Cohort

Number of Patients


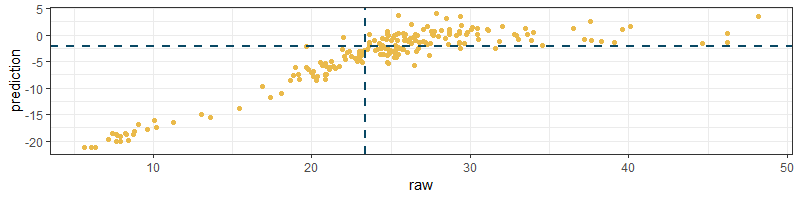


Predicted Violation

Planned Contralateral Parotid Gland Dmean

Figure S.7: Candidate ART inclusion/exclusion cutoff values for the simplified patient selection guidelines relative to cohort data and model predictions for the high-importance predictor: planned contralateral parotid gland Dmean dose. *Top:* Reference histogram of planned contralateral parotid gland Dmean doses across the cohort. Vertical dashed line denotes the candidate inclusion/exclusion cutoff value of 24 Gy. *Middle:* Histogram of candidate cutoff values across the cohort. The median candidate cutoff was rounded to 24 Gy for practicality (2^nd^ percentile). *Bottom:* distribution of planned contralateral parotid gland Dmean versus numerical model predictions of objective violations. The horizontal dashed line denotes the ROC-based threshold of -2.2 Gy used to convert numeric model predictions into “normal” vs. “violation” classifications. Vertical dashed line denotes the inclusion/exclusion cutoff value of 24 Gy. Datapoints in the upper right quadrant are “true positives”.


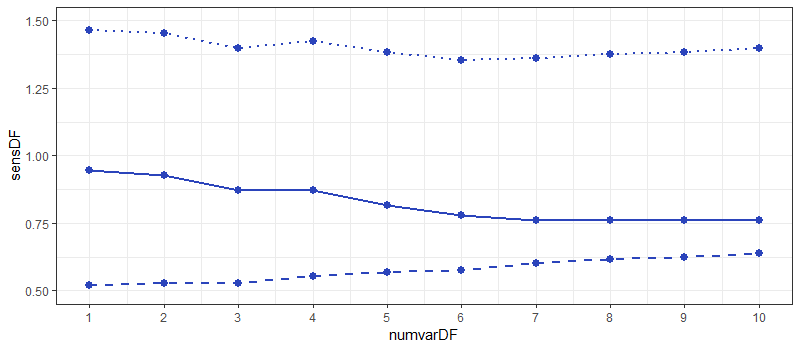


Legend: Sensitivity Specificity Youden Index

Number of Variables

Performance

Figure S.8: Changes in patient selection guideline performance with number of variables included. Sensitivity and specificity was comparable for guidelines comprised of 6 to 10 variables. A final guideline with 8 variables was selected as the addition of the 9^th^ and 10^th^ variable only provided negligible increases in Youden index.

**Supplementary References:**

Brock KK, Mutic S, McNutt TR, Li H, Kessler ML. Use of image registration and fusion algorithms and techniques in radiotherapy: Report of the AAPM Radiation Therapy Committee Task Group No. 132: Report. *Med Phys*. 2017;44(7):e43-e76.

Hansen CR, Christiansen RL, Lorenzen EL, et al. Contouring and dose calculation in head and neck cancer radiotherapy after reduction of metal artifacts in CT images. *Acta Oncol*. 2017;56(6):874-878.

Lim TY, Gillespie E, Murphy J, Moore KL. Clinically Oriented Contour Evaluation Using Dosimetric Indices Generated From Automated Knowledge-Based Planning. *Int J Radiat Oncol Biol Phys*. 2019;103(5):1251-1260.

Weppler S, Schinkel C, Kirkby C, Smith W. Data clustering to select clinically-relevant test cases for algorithm benchmarking and characterization. *Phys Med Biol*. 2020a;65(5):1-12.

Weppler S, Schinkel C, Kirkby C, Smith W. Lasso logistic regression to derive workflow-specific algorithm performance requirements as demonstrated for head and neck cancer deformable image registration in adaptive radiation therapy. *Phys Med Biol*. 2020b:65(19):1-12.
